# Virus-Shaped Mesoporous Silica Nanostars to Improve the Transport of Drugs across the Blood–Brain Barrier

**DOI:** 10.1021/acsami.4c06726

**Published:** 2024-07-11

**Authors:** Alessandra Pinna, Ieva Ragaisyte, William Morton, Stefano Angioletti-Uberti, Alizé Proust, Rocco D’Antuono, Chak Hon Luk, Maximiliano G. Gutierrez, Maddalena Cerrone, Katalin A. Wilkinson, Ali A. Mohammed, Catriona M. McGilvery, Alejandro Suárez-Bonnet, Matthew Zimmerman, Martin Gengenbacher, Robert J. Wilkinson, Alexandra E. Porter

**Affiliations:** † School of Veterinary Medicine, Faculty of Health and Medical Sciences, University of Surrey, Guildford GU2 7XH, U.K.; ‡ The Francis Crick Institute, NW1 1AT London, U.K.; § Department of Materials, Imperial College London, SW7 2AZ London, U.K.; ∥ Dyson School of Design Engineering, Imperial College London, SW7 2AZ London, U.K.; ⊥ Centre for Infectious Diseases Research in Africa (CIDRI-Africa), Institute of Infectious Disease and Molecular Medicine, University of Cape Town, Observatory, Cape Town 7925, Republic of South Africa; # Department of Medicine, University of Cape Town, Observatory, Cape Town 7925, Republic of South Africa; ∇ Department of Infectious Diseases, Imperial College London, W12 0NN London, U.K.; ○ Crick Advanced Light Microscopy STP, The Francis Crick Institute, NW1 1AT London, U.K.; ◆ School of Design, Royal College of Art, SW11 4AY London, U.K.; ¶ Department of Biomedical Engineering, School of Biological Sciences, University of Reading, Reading RG6 6AY, U.K.; ⋈ Department of Pathobiology and Population Sciences, The Royal Veterinary College, North Mimms, Hatfield, Hertfordshire AL9 7TA, U.K.; ⧓ Center for Discovery and Innovation, Hackensack Meridian Health, 111 Ideation Way, Nutley, New Jersey 07110, United States; ⧖ Hackensack Meridian School of Medicine, Nutley, New Jersey 07110, United States

**Keywords:** mesoporous silica nanoparticle, brain diseases, blood–brain barrier model, molecular dynamics simulation, nanostar

## Abstract

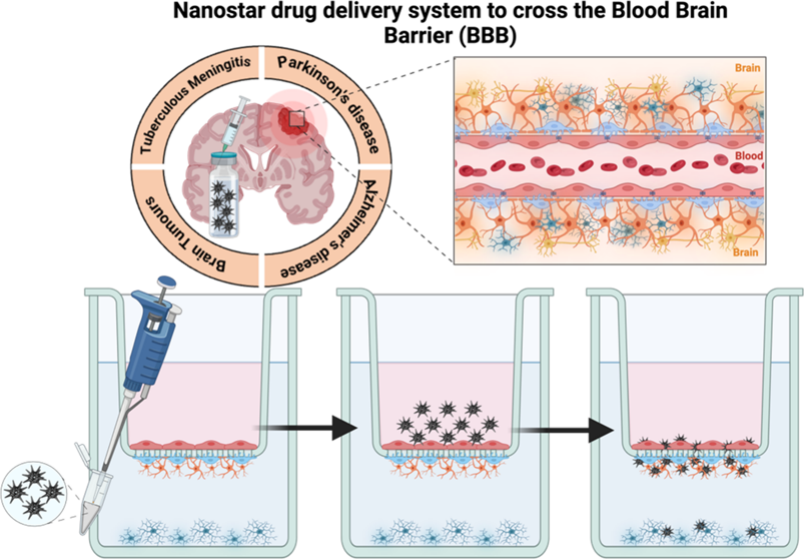

Conditions affecting the brain are the second leading cause of death globally. One of the main challenges for drugs targeting brain diseases is passing the blood–brain barrier (BBB). Here, the effectiveness of mesoporous silica nanostars (MSiNSs) with two different spike lengths to cross an *in vitro* BBB multicellular model was evaluated and compared to spherical nanoparticles (MSiNP). A modified sol–gel single-micelle epitaxial growth was used to produce MSiNS, which showed no cytotoxicity or immunogenicity at concentrations of up to 1 μg mL^–1^ in peripheral blood mononuclear and neuronal cells. The nanostar MSiNS effectively penetrated the BBB model after 24 h, and MSiNS-1 with a shorter spike length (9 ± 2 nm) crossed the *in vitro* BBB model more rapidly than the MSiNS-2 with longer spikes (18 ± 4 nm) or spherical MSiNP at 96 h, which accumulated in the apical and basolateral sides, respectively. Molecular dynamic simulations illustrated an increase in configurational flexibility of the lipid bilayer during contact with the MSiNS, resulting in wrapping, whereas the MSiNP suppressed membrane fluctuations. This work advances an effective brain drug delivery system based on virus-like shaped MSiNS for the treatment of different brain diseases and a mechanism for their interaction with lipid bilayers.

## Introduction

The brain is considered the most complex organ in the human body influencing every aspect of life. According to the World Health Organization (WHO), in 2022, conditions affecting the brain and nervous system, such as neurodegenerative disorders, cerebrovascular diseases, neuroinfectious disorders, and cancer, are the second leading cause of death globally, with about 9 million deaths per year.^1^ There are still many unmet medical needs related to the treatment of most neurological diseases. One major limitation in delivering drugs into the brain arises due to the impermeability of the blood–brain barrier (BBB) in the neurovascular system. The BBB is made up of specialized endothelial cells that are connected *via* tight junction complexes that enclose the capillaries in the brain. The layer of endothelial cells is also supported and maintained by pericytes and astrocytes.^2,3^ This organized cell structure is highly selective, resulting in a highly controlled permeability of the BBB. Crossing the BBB requires molecules to be small (<500 Da) and have no more than 8–10 hydrogen bonds (partially lipophilic).^4^ Most brain-targeting drugs do not cross the BBB despite being lipid-soluble small molecules. Of the 8000 drugs reported in the Comprehensive Medicinal Chemistry database, only 6% are active in the brain and can treat a limited group of brain diseases.^5^ There is an urgent clinical need for alternative technologies to deliver drugs across the BBB to treat brain diseases locally, avoiding the shortcomings associated with current treatments.

Nanostructures have promise to increase the brain bioavailability of many drug molecules. Their many unique characteristics, including their small hydrodynamic diameter, cell selectivity, low toxicity, biodegradability, and solubility,^6^ have all been utilized to create “designer” nanoparticles (NP) for drug delivery through BBB *via* nondisruptive pathways.^7^ Overall, a wide range of NP types, including both organic and inorganic, as well as different BBB penetration mechanisms have been investigated, each with their own advantages and limitations.^8,9^ Mesoporous silica NP (MSiNP) are of great interest as drug delivery vehicles and a theranostic platform due to their large and tunable porosity, high surface area, low toxicity, and controllable sizes.^10−12^ Previous work has demonstrated that spherical MSiNP can penetrate the BBB. For instance, Chen et al. used 50 nm negatively charged MSiNP to release doxorubicin into the larval zebrafish brain and showed that MSiNP-mediated BBB penetration is charge-dependent, and no NP were detected in the zebrafish brain when the MSiNP’s size was increased from 50 to 200 nm.^13^ Mohammadpour et al. demonstrated that intravenously administered MSiNP in mice do not produce any pathologic lesions or chronic toxicity after one year *in vivo* when compared to dense silica NP.^14^ Their high surface area and limited condensation of the siloxane framework promote silica dissolution into nontoxic, soluble silicic acid species.

The shape of NP plays a key role in the NP transport across the BBB.^15,16^ Nowak et al. demonstrated that the transport rate of polystyrene rods was about twice that of spheres in a microfluidic chip BBB model, with rod-shaped particles showing better transport across the BBB.^17^ Mimicking viruses in size, shape, and surface properties can facilitate NP uptake into cells, including neuronal types.^18−20^ Moreover, Wang et al. showed that virus-like MSiNP have high drug loading, longer circulation times, as well as higher internalization within HeLa and RAW264.7 cells.^21^ We have also shown that L-dopa-functionalized gold nanostars cross an *in vitro* model of the three-dimensional (3D) human-derived brain endothelial cells.^22^ Hence, flower-, star-, and virus-shaped NP can enhance translocation across the BBB and maximize drug delivery within the body, specifically inside the brain. Virus-shaped mesoporous silica nanostructures have not previously been trialed as vectors for drugs to cross the BBB.

Different types of BBB *in vitro* coculture models have been used to test the ability of drugs and nanocarriers to cross the BBB.^23−25^ The most common model uses a transwell insert, which mimics the blood (apical) side, while the well in which the insert fits mimics the brain (basolateral) side,^26^ with the microporous membrane (0.4–3 μm) between the two compartments. More recently, a microfluidic-based *in vitro* BBB model has been introduced to screen the BBB permeability of new drugs *in vitro*.^27,28^ Coculture and multicellular systems that include endothelial cells, astrocytes, and pericytes more closely model the organization of the BBB *in vivo* as the cells are in direct contact and promote the exchange of growth factors required for cellular growth and development.^29,30^ One-cell and three-cell type BBB models have successfully been used to measure NP transport across the BBB.^31^ An optimal representation of the *in vivo* BBB would be to have a multicell culture model including neurons and microglia, which contribute to the maintenance of the BBB and, together with endothelial cells, astrocytes, and pericytes, form the neurovascular unit (NVU).^25^ Another crucial aspect of developing an *in vitro* BBB model is the choice of the cell source. Primary cells derived from human tissues are often regarded as the optimal choice for the model due to their closer resemblance to the biological properties of the *in vivo* BBB.^32^ To the best of our knowledge, a multicell culture BBB *in vitro* model, based on four cell types (two primary cells and two cell lines), has never been used to test the mesoporous silica nanostars’ (MSiNS) ability to cross the BBB.

**Figure 1:**
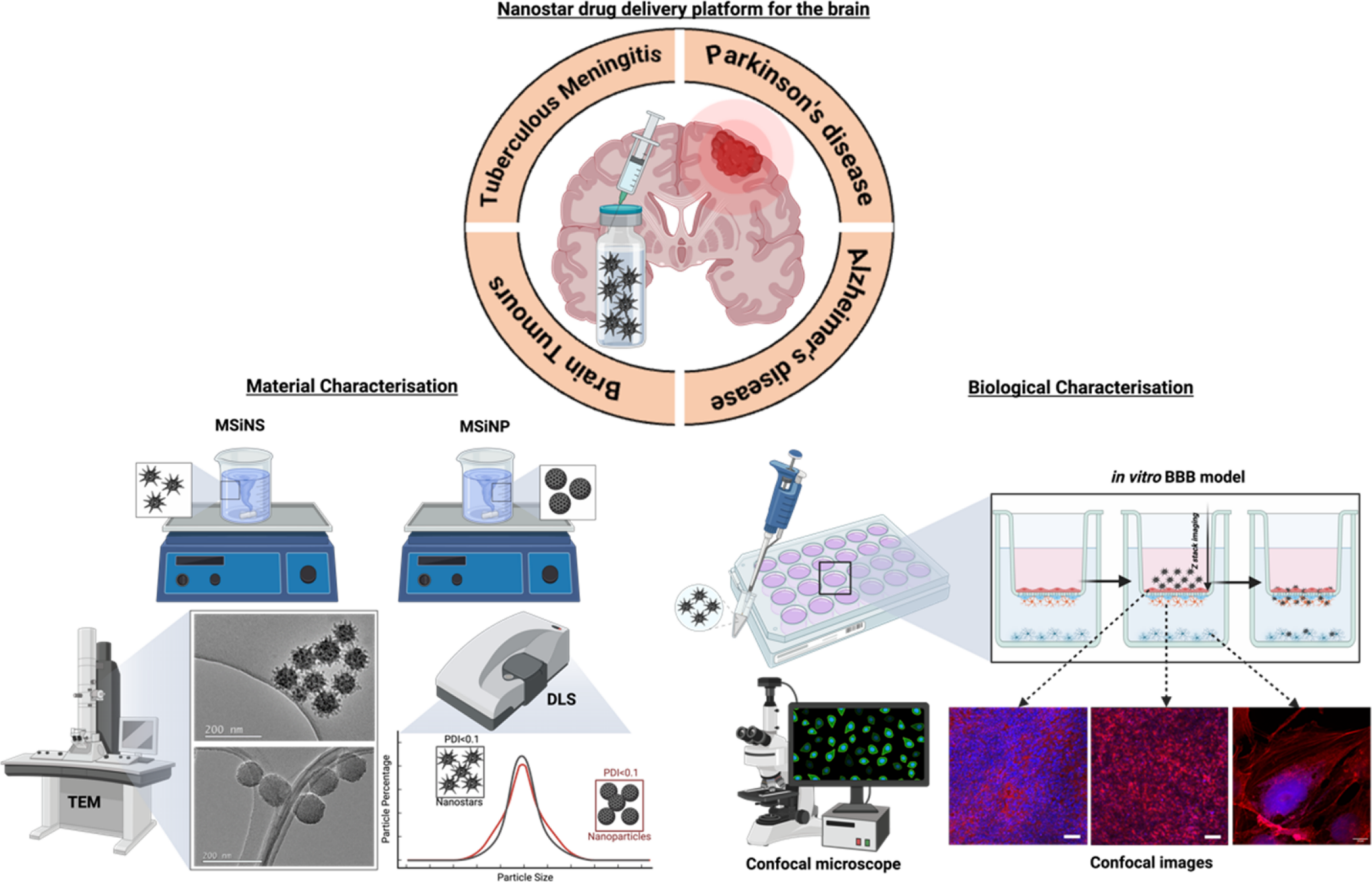
Schematic diagram of the virus-like MSiNS drug delivery system (top). Production and characterization (left) and their evaluation in the *in vitro* coculture/multicell BBB model (right).

This work elucidated whether the uptake and transport of MSiNS through an *in vitro* human tissue-derived BBB coculture/multicellular transwell model could be controlled by tuning their spike length (Figure 1). Two types of nanostars with shorter (MSiNS-1) and longer (MSiNS-2) spikes were chosen to study the influence of the spike length on uptake into the BBB model and BBB penetration. We hypothesized that the presence of spikes on the nanostar surface makes the cell membranes more weakly pinned to a nanostar’s surface compared to that of spherical nanoparticles, which makes it easier for the membrane to rearrange and wrap around the nanostar, resulting in enhanced uptake, translocation, and release into the basolateral side of the *in vitro* BBB model.

## Materials and Methods

### Chemicals

(3-Aminopropyl)triethoxysilane (APTES, 99%, Sigma-Aldrich), 5/6-carboxyfluorescein succinimidyl ester (NHS-Fluorescein, Thermo Fisher Scientific, Germany), acetone (≥99.0% Ph. Eur., VWR), cyclohexane (anhydrous, 99.5%, Sigma-Aldrich, Germany), distilled water, absolute ethanol (≥99.5% Ph. Eur., VWR), hexadecyltrimethylammonium bromide (CTAB, >97%, Merck KGaA, India), *n*-hexane (≥97%, for HPLC, VWR, EC), *N*,*N*-dimethylformamide (DMF, anhydrous, 99.8%, Sigma-Aldrich, Germany), sodium hydroxide (NaOH, 99%, VWR Chemicals, Belgium), and tetraethyl orthosilicate (TEOS, ≥99.0%, Sigma-Aldrich, Germany) were used, as received, without further purification.

### Mesoporous SiO_2_ Nanostar (MSiNS) Synthesis

MSiNS were synthesized using a modified epitaxial growth approach previously reported by Wang et al.^21^ Briefly, a cetyltrimethylammonium bromide surfactant (CTAB, 0.25–1.00 g) was mixed with deionized water (50 mL) and a base catalyst (0.8 mL, 0.1 M NaOH (aq)) and stirred for 2 h at a synthesis temperature of 50–70 °C. Then, a silica source, tetraethyl orthosilicate (TEOS, 4 mL), was mixed with the organic cosolvent (cyclohexane or *n*-hexane, 16 mL) and added slowly to the reaction mixture. Following the addition, the reaction was left to age for 72–96 h under constant stirring (150–300 rpm). The reaction mixture was centrifuged (40 min. 7830 rpm), and the pellets were washed with ethanol (30 mL) once and deionized water (30 mL) twice, with the pellets dispersed completely under sonication during each wash. The pellets were dried overnight at 60 °C, calcinated at 550 °C (6 h, 3 °C/min increase) to remove any leftover surfactant, and ground into a powder.

### Mesoporous SiO_2_ Nanoparticle (MSiNP) Synthesis

MSiNP were synthesized using a modified sol–gel Stöber method reported in our previous work.^33^ Briefly, 1 g of CTAB was dissolved into 500 mL of distilled water in a 1 L round-bottom flask and stirred until the solution became clear. The flask was immersed in paraffin oil at 70 °C and 600 rpm. Then, 3 mL of a 2 M NaOH catalyst was added to the CTAB solution. After 5 min, 5 mL of TEOS was added dropwise, followed by the addition of 5 mL of cosolvent ethyl acetate after 1 min. The solution was aged for 1 h with stirring (600 rpm) and further 3 h without stirring at 70 °C. The MSiNP powder was washed 3 times in ethanol and centrifuged at 7830 rpm for 40 min. Next, the MSiNP were dried overnight at 60 °C. Finally, the CTAB was eliminated in a furnace at 550 °C for 6 h at a heating rate of 3 °C/min.

### FITC Functionalization of MSiNS and MSiNP

The MSiNS and MSiNP surfaces were first functionalized with 3-aminopropyltriethoxysilane by mixing 200 mg of MSiNS or MSiNP with 3 mL of APTES and 50 mL of EtOH at room temperature under stirring. The amino-functionalized NPs (NH_2_-MSiNS, NH_2_-MSiNP) were washed with EtOH and centrifuged at 7830 rpm. Then, 4 mg of NH_2_-MSiNS/NH_2_-MSiNP were resuspended in 0.5 mL of DMF, mixed with 0.5 mM 2 mg mL^–1^ FITC in DMF, and stirred at room temperature for 4 h. The FITC-NH_2_-MSiNS/FITC-NH_2_-MSiNP were washed once with acetone and twice with water and centrifuged at 7830 rpm. The obtained precipitate was dried overnight at 60 °C.^34^

### Physicochemical Characterization

Transmission electron microscopy (TEM) was performed using a JEOL 2100Plus 200 kV TEM (Tokyo, Japan) equipped with a Gatan CCD camera (Pennsylvania). The samples for TEM were prepared as 10 ppm suspensions in isopropanol, cast on a 300-mesh copper grid with a holey carbon film, and left to air-dry. TEM images were analyzed using Fiji.^35^ The images were first converted to binary format, and then the “fill holes” function was used to remove the white space at the center of the particles caused by sample porosity. Following that, the “watershed” function was used to separate particles where more than one particle is present in the image. Finally, the “analyse particles” function was used to obtain the area of each particle in squared pixels, which was then converted to the particle radius in nanometers, assuming a spherical particle of an equivalent area. The spike length was measured by manually selecting 50 spikes and drawing a line alongside each spike, and then converting the length of each line into nanometres.

Electron tomography was performed on a JEOL 2100F TEM (Tokyo, Japan) in scanning transmission electron microscopy (STEM) using a high-angle annular dark field detector (HAADF) operated at 200 kV. In a HAADF image, the intensity is proportional to the atomic number squared (*Z*^2^) of the elements within a feature. The tomography data series was acquired using Serial EM.^36^ On controlling the tilt series acquisition, using the “low dose” mode allowed electron beam damage effects to be minimized. Tilt series were acquired between −70 and 70° at 2° intervals. No change in the structure of the MSiNS was observed between the first and last images in the data set, indicating that minimal electron beam damage had occurred. Tomographic data sets were aligned using Inspect3D (Thermo Fisher Scientific).

Dynamic light scattering (DLS) and ζ-potential measurements were collected using a Malvern ZetaSizer Nano ZSP (Malvern, U.K.) by dispersing the nanoparticles in 10 ppm deionized water solutions at pH 7. The solutions were sonicated prior to measurement to disperse the particles. Disposable plastic cuvettes were used for DLS measurements, and disposable folded capillary cells (DTS1070, Malvern, U.K.) were used for ζ-potential measurements. Data were acquired in triplicate for all samples.

### Human Cell Culture Prior to BBB Assembly

Human fetal astrocytes were isolated from the fetal brain, provided by the Joint MRC/Wellcome Human Developmental Biology Resource with ethical approval (University College London, UCL, site REC reference: 18/LO/0822-IRAS project ID: 244325 and Newcastle site REC reference: 18/NE/0290-IRAS project ID: 250012), as previously described.^37,38^ In short, blood vessels and meninges were removed from the fetal brain tissue (15–20 gestational weeks). Before being passed through a 70 μm cell strainer (Corning), the tissue was minced, treated with 0.2 mg/mL DNase I (Sigma-Aldrich) and 0.25% trypsin (Thermo Fisher Scientific) for 30 min. The flow-through was plated in Petri dishes for adherent cells (Sarstedt) at a final concentration of (6–8) × 10^7^ cells/Petri in MEM supplemented with 10% FBS, 100 U/ml penicillin, 100 μg/mL streptomycin, 0.3 mg/mL l-glutamine, 1 mM sodium pyruvate, 1× MEM nonessential amino acids, 0.5 μg/mL amphotericin B, and 2.5 mL of a glucose solution (all from Thermo Fisher Scientific). Cells were then maintained in Dulbecco’s modified Eagle’s medium (DMEM; corning) supplemented with 10% fetal bovine serum (FBS) (Thermo Fisher Scientific) and 1× penicillin–streptomycin (Thermo Fisher Scientific).

Primary human brain vascular pericytes (HBVP) were commercially obtained (ScienCell Research Laboratories). Cells were cultured in a pericyte medium (PM, ScienCell Research Laboratories) supplemented with 1× penicillin–streptomycin (Thermo Fisher Scientific) in a flask previously coated with (2 μg/cm^2^) poly-l-lysine (Sigma-Aldrich) to promote cell adhesion.

Human microglial cells (HMC3) were obtained commercially (ATCC CRL3304) and cultured in Eagle’s minimum essential medium (EMEM; ATCC) supplemented with 10% FBS (Thermo Fisher Scientific) and 1× penicillin–streptomycin (Thermo Fisher Scientific).

Human cerebral microvascular endothelial cells (hCMEC/D3), derived from human temporal lobe microvessels isolated from tissue excised during surgery for control of epilepsy, were commercially obtained (Merck). Cells were cultured in EndoGRO-MV Complete Media Kit (Millipore) supplemented with (1 ng mL^–1^) human recombinant FGF-2 basic protein (Millipore) in a flask previously coated with (150 μg mL^–1^) collagen I (Sigma-Aldrich) to promote cell adhesion. All types of cells were maintained at 37 °C in a humidified incubator with 5% CO_2_ and used at up to 6 passages for the experiments.

### Assembly and Coculturing of the Human-Derived Cells in the *In Vitro* BBB Model System

An advanced *in vitro* model^37^ was used, which resembles the anatomical structure of the BBB *in vivo*, including hCMEC/D3 cells and a mix of HBVP and astrocytes seeded on each side of a porous insert. This configuration enables cell–cell physical contact with astrocytes projecting their end feet toward hCMEC/D3 cells through the porous membrane. Moreover, microglia were seeded on the bottom of the well to mimic their localization in the central nervous system (CNS). This *in vitro* BBB model was prepared using cell culture inserts for 24-well plates with a 3.0 μm pore translucent PET membrane (Corning Life Sciences). The upper side of the inset was coated with (150 μg mL^–1^) collagen I (Sigma-Aldrich).

A mix of HBVP (10^4^/insert) and astrocytes (5 × 10^4^/insert) was then seeded on the basolateral side of the membrane, and cells were allowed to adhere for at least 4 h in the incubator. Soon after, the hCMEC/D3 cells (2.5 × 10^4^/insert) were seeded on the apical side of the membrane.

Cells were allowed to grow in 150 μL (apical chamber) and 750 μL (collector) of BBB medium 1, composed of a 50% hCMEC/d3 medium and a 50% HBVP medium, for 6–7 days to reach confluence. The media in both the apical chamber and collector were refreshed with 150 and 750 μL of BBB medium 1, respectively, on day 4. HCM3 (5 × 10^3^/well) cells were then seeded at the bottom of the well containing the inserts, and media were changed (apical chamber and collector) to a mix of a 50% hCMEC/D3 medium, a 25% HBVP medium, and a 25% HMC3 medium (BBB medium 2).

### BBB Model Integrity Assessment

The BBB integrity was assessed by measuring its permeability to dextran–rhodamine B. Briefly, the culture medium in the upper chamber was replaced with BBB medium 2 supplemented with 0.5 mg mL^–1^ 70 kDa dextran–rhodamine B (Life Technologies). After 4–5 h, the fluorescence intensity in the collector was measured using a Synergy 2 multimode microplate reader (Biotek). Samples displaying dextran–rhodamine B permeability greater than 20% of the empty control insert were discarded.

### MSiNS Cytotoxicity Assay

Astrocyte, HBVP, HMC3, and hCMEC/D3 cells were seeded in a monoculture at a concentration (5 × 10^4^ cells mL^–1^) in a flat-bottomed 96-well plate and incubated at 37 °C under 5% CO_2_ for 24 h to allow the cells to attach in a monolayer and expand. The 96-well plate was previously coated with poly-l-lysine (pericytes and astrocytes) or collagen type I (hCMEC/d3).

The cells were exposed to MSiNS at a concentration range from 0 to 1000 (0, 0.0001, 0.001, 0.01, 0.1, 1, 10, 100, 1000 μg mL^–1^) for 24, 48, and 72 h. Cell viability was then assessed using an MTS colorimetric assay (The CellTiter 96, Promega), which contains a tetrazolium compound [3-(4,5-dimethylthiazol-2-yl)-5-(3-carboxymethoxyphenyl)-2-(4-sulfophenyl)-2*H*-tetrazolium, inner salt; MTS] and an electron-coupling reagent (phenazine ethosulfate; PES).

Further, 20 μL of a CellTiter 96 reagent was added directly to cultured wells, incubated for 1 h, and then the concentration of the formazan product, which is directly proportional to the number of living cells in culture, was recorded at 490 nm using a microplate reader (TriStar^2^ LB 942 Multimode Microplate Reader).

### *In Vitro* Cytotoxicity and Immunogenicity MSiNS Test

Cryopreserved peripheral blood mononuclear cells (PBMC) isolated from leukocyte cones obtained from healthy donors (NHS Blood and Transplant) were thawed and rested in RPMI 1640 (Thermo Fisher Scientific, Gibco) containing 10% heat-inactivated FCS (Thermo Fisher Scientific, Gibco) and 1% penicillin–streptomycin (Thermo Fisher Scientific). Viability was assessed using Trypan blue on an automated cell counter. Only samples with viability greater than 90% were used for the experiments. A total of 2 × 10^4^ PBMC and MSiNS at a concentration range from 0 to 1000(0, 0.0001, 0.001, 0.01, 0.1, 1, 10, 100, 1000 μg mL^–1^) were added to each 96-well plate well and incubated for 24, 48, and 72 h at 37 °C. The MTS colorimetric assay (The CellTiter 96, Promega) was used to assess cell viability at each time point. Following centrifugation, cell culture supernatants were collected for the quantification of analytes. A custom Milliplex multiplex assay (Merck) was used to measure proteins on the Bio-Plex platform (Bio-Rad Laboratories) using Luminex xMAP. Measured analytes included GM-CSF, IFN-γ, IL-1β, IL-2, IL-4, IL-6, IL-10, IL-12p70, and TNF. All assays were conducted according to the manufacturer’s recommendations. Staphylococcus enterotoxin B (SEB), which promotes cytokine release and inflammation, was used as a positive control.

### BBB Translocation Assay for MSiNS

Nanoparticles were prepared by resuspending in BBB medium 2 and adding to the cultured BBB at the concentration of 1 μg mL^–1^. At 24-, 48-, and 96 h postaddition of MSiNS/MSiNP, the BBB was harvested and fixed. The chemical inhibitors latrunculin A (Thermo Fisher), dynasore hydrate (Merk), and amiloride hydrochloride (VWR) were administered at concentrations of 80, 80, and 40 μM, respectively. Throughout the course of the experiment, the cells comprising the BBB model were treated with these inhibitors for 30 min prior to the addition of the MSiNS/MSiNP.

### Fixation and Immunofluorescence Staining

The cultured BBB were washed once with PBS (1×) and fixed in 4% PFA in PBS for 30 min. The fixed BBB were then washed 3 times with PBS and permeabilized with 0.1% Triton + 2%BSA + PBS for 5 min. The permeabilized BBB were washed twice with PBS and stained with AlexaFluor 647 Mouse anti-GFAP (BD) for 1 h, washed 3 times with PBS, stained with Cytopainter F-actin (Abcam) for 30 min, washed 3 times with PBS, and stained with DAPI (Merk) for 10 min. The stained BBB were washed 3 times with PBS and mounted between glass coverslips (Thermo Fisher) and microscope slides, Superfrost (VWR), using a mounting agent (Merk), and dried overnight at room temperature.

### Confocal Microscopy

A Zeiss LSM880 confocal microscope equipped with a 20×/0.8 NA objective was used to acquire a 20 μm Z-stack centered around the position of the transwell membrane with a z-step of 0.5 μm. The data sets were acquired using the sequential acquisition of 4 channels: DAPI (cell nuclei), FITC (MSiNS or MSiNP), AlexaFluor 647 (Actin), and transmitted light (membrane). Images of MSiNS’s uptake into PBMC cells were acquired on a Zeiss LSM880 with a 40×/1.1 NA water immersion objective (zoom factor 2×), with a pixel size of 100 nm. An orthogonal view of a z-stack was acquired on a Zeiss LSM880 with a 20×/0.8 NA dry objective (zoom factor 4×), with a pixel size of 100 nm and a z-step of 500 nm.

### Image Analysis

The acquired images were analyzed in Fiji, measuring the mean fluorescence intensity of the overall image in the FITC channel (NP) along the z-stack using the command “Measure Stack”. Before the MFI measurements, the z-stacks of individual positions were processed to subtract the background due to the porous membrane (original image minus a Gaussian blur of sigma σ = 1 of the same stack). Finally, the MFI values of each slice of the z-stack were plotted, assuming that the z-stacks were centered around the position where the membrane pores were in focus.

### Animal Studies and Histopathology Examination

All animal studies were reviewed and approved by the Hackensack Meridian Health Institutional Animal Care and Use Committee. Further, 8–10-week-old female CD-1 mice (Charles River Laboratory) with access to food and water *ad libitum* were maintained under specific pathogen-free conditions. Animals received a single dose of MSiNS-1 at 10 mg/kg formulated in 0.5% methyl cellulose/water *via* the lateral tail vein. At 3, 7, and 24 h postdosing, groups of 3 animals were euthanized for quantification of nanostars in whole blood and brain by using inductively coupled plasma mass spectrometry (ICP-MS) spectroscopy.^39^

Brain tissues were dried at 50 °C overnight to obtain the dry weight, followed by digestion in an oven at 70 °C overnight using a 0.4 mL HNO_3_–0.1 mL H_2_O_2_ mixture and then diluted to a total volume of 8 mL using purified water. Blood samples were prepared by digesting 0.1 mL in an oven at 70 °C overnight using a 0.3 mL HNO_3_–0.1 mL H_2_O_2_ mixture and diluting to a total volume of 6 mL using purified water. All samples were prepared in acid-cleaned 15 mL centrifuge tubes (HDPE trace metal grade tubes; Elkay Scientific). Each sample was spiked with the Rh internal standard solution to obtain a concentration of 50 μg/L. All silica quantification was conducted on a PerkinElmer NexION 350D inductively coupled plasma quadrupole mass spectrometer (ICP-QMS) under kinetic energy discrimination (KED) mode at the London Metallomics Facility, King’s College London.

Tissue samples of the brain were fixed overnight in 10% formalin and embedded in paraffin (FFPE). Brain sections of 3–4 mm mounted on glass slides were subjected to hematoxylin and eosin (HE) staining prior to imaging using a Panoramic Desk slide scanner (3D Histech) and examined by a board-certified veterinary pathologist (ASB). Histopathological assessment was performed blind to experimental grouping using a light microscope (Olympus BX43). Tissue sections were examined to assess any morphological evidence of inflammation, tissue degeneration, and/or necrosis.

### Statistical Analysis

Statistical analysis was performed using GraphPad Prism software version 10. Statistical details of each experiment (statistical tests used, exact value of *n*, dispersion, and precision measures) can be found in the figure legends. Statistical analyses were performed on raw data for all experiments using a two-way analysis of variance (ANOVA) with correction for multiple comparisons (Dunnett). A significance level of * *p* < 0.05 was considered statistically significant. ** *p* < 0.01, *** *p* < 0.001, and ****P < 0.0001 were deemed highly significant when compared with control groups. When nonstatistically significant, no star has been added to figures for the sake of clarity.

### Modeling MSiNS/MSiNP Cell Interactions

The uptake mechanisms of MSiNS and MSiNP were simulated *via* molecular dynamics (MD) using the LAMMPS package.^40^ Both nanoparticles and the lipid bilayer membrane were represented using a coarse-grained model. We present this model using Lennard-Jones units, where ε and^41^ τ are the fundamental units for energy, length, and time, respectively. These units can be later mapped onto real units by comparing them to the experimental system, as shown later in the paper. Regarding the coarse-grained model, for the lipid bilayer, a widely used and validated 3-bead parametrization was employed, commonly known as the implicit-solvent Cooke–Deserno model.^41^ Of the three beads, one represents the lipid head, and two beads are used to represent the lipid tail. Each of the three beads is connected by a finite extensible nonlinear elastic (FENE) bond. The equation for the FENE bonds is reproduced below in eq 1, where *r* is the distance between two beads, *r*_0_ is the equilibrium bond length of 1.5σ, and *K*_1_ is the spring constant of 30 ε.
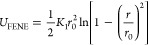
1

The angle between the beads, θ, is maintained at 180, θ_0_, by a harmonic bond, shown in eq 2, between all three beads, with a spring constant, *K*_2_, of 10 ε.
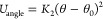
2

Nonbonded interactions between the beads describing the lipids are described using the following two equations, the parameters of which are reported in Table 1. The Weeks–Chandler–Anderson potential in eq 3 is used to alter the relative exclusion radius, or size, of each bead. The cosine potential in eq 4 accounts for the attraction between lipid tails, which enables membrane formation.
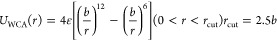
3
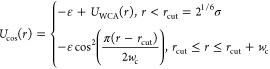
4

**Table 1:** Nonbonded Interaction Parameters between Bead Types in the Simulated Nanoparticle–Membrane System

bead type	bead type	interaction	parameter
lipid head	lipid head	*U*_WCA_	*b* = 0.95σ
lipid head	lipid tail	*U*_WCA_	*b* = 0.95σ
lipid tail	lipid tail	*U*_cos_	*b* = σ, *w*_c_ = 1.5σ
nanoparticle	lipid head	*U*_WCA_	*b* = σ
nanoparticle	lipid tail	*U*_WCA_	*b* = σ
ligand	lipid head	*U*_WCA_	*b* = σ
ligand	lipid tail	*U*_WCA_	*b* = σ
nanoparticle	ligand	-	-
receptor	ligand	*U*_WCA_	*b* = σ
receptor	lipid head	*U*_WCA_	*b* = 0.95σ
receptor	lipid tail	*U*_WCA_	*b* = 0.95σ
receptor	nanoparticle	*U*_WCA_	*b* = σ

The temperature within the simulation was kept constant at 1 ε/*k*_B_, where *k*_B_ is the Boltzmann constant, using a Langevin thermostat with a dampening constant of 1τ^–1^. Using the coarse-grained model previously described, a square bilayer was built using 10,452 lipids totaling 77σ × 77σ in length along the *X* and *Y* dimensions when equilibrated with the Weeks–Chandler–Anderson cutoff of 1.5σ.^42^ Zero tension was imposed in the *XY* plane of the membrane using a Nose–Hoover barostat with a pressure-dampening constant of 1τ. Each system was simulated five times for 10^4^τ, with a time step of 0.01τ.

The membrane’s thickness was determined by projecting the lipid heads from the top and bottom leaflet onto a surface divided into equally sized bins of width 0.5σ in the *XY* plane. Empty grid spaces were filled using the linear interpolation package from SciPy.^43^ Leaflets were assigned based on the angle of the normal vector that aligns the tail’s last bead to the lipid head. The *Z* position of each head within a bin was then averaged, and the thickness was calculated as the average difference between the *Z* positions in the leaflets for each bin.

A previously published method was modified to generate the MSiNS/MSiNP surfaces, ensuring that each bead (representing the surface) was evenly spaced to achieve a homogeneous surface.^44^ An explanation of the algorithm can be found in the SI Appendix. The nonbonded interaction between MSiNS beads and lipids, ligands, and receptors utilizes the same potentials as nonbonded interactions between lipids in eqs 3 and 4. The parameters for these interactions can be seen in Table 1.

The MSiNS/MSiNP were scaled down from their synthesized size to reduce the length of simulations. MSiNS were generated with a radius of 7σ, a tip length of 5σ, a basal tip radius of 3σ, and a tip radius of 1.5σ. MSiNP were modeled as spherical particles with a radius of 12σ.

Endocytosis was simulated by assuming that cells can internalize all tested MSiNS/MSiNP through ligand–receptor interactions.^45^ In the membrane, 50% of the lipid heads were treated as receptors, whereas a variable number of nanoparticle surface beads were turned into ligands to investigate the effect of their surface concentration as well as their spatial distribution, as described later in more detail. The attractive interactions between ligands and receptors are modeled using the Morse potential, as shown in eq 5.
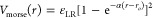
5

A dissociation energy of ε_LR_ = 30 ε is used, with an equilibrium length of *r*_o_ = 1 σ and α = 1 σ. A valence constraint was enforced to ensure that each ligand could only interact with one receptor at a time by creating reversible Morse bonds between ligands and receptors. More precisely, we form bonds with a 50% chance when an unbound ligand and an unbound receptor are within 1.3σ from each other. Instead, a bond breaks (with 100% probability) if the bond length exceeds 4σ.^46^ Ligands were only placed on the tips of MSiNS, covering the outermost 3σ of the maximum radius (12σ). By only placing ligands on a fraction of the tips, rather than randomly distributed on the entire surface, we more closely represent possible steric restrictions of ligand binding.^47−49^ Additionally, the high curvature and small volume closer to the MSiNS core may be inaccessible to the membrane. The overall goal was to ensure that the MSiNS and MSiNP had the same number of accessible ligands so that the impact of particle shape could be isolated. The independent variable considered throughout the simulated study was the number of tips on the MSiNS, as more tips meant more ligands. Therefore, the comparative spherical MSiNP will have the same number of ligands evenly distributed in patches around the surface. The total number of patches equals the number of tips on the comparative MSiNS.

During our simulations, we observe that once the MSiNS/MSiNP and the membrane are in contact, the latter starts to wrap around the MSiNS/MSiNP surface. The wrapping fraction is a widely used metric to compare NP uptake in molecular dynamics simulations, indicating the extent to which the NP surface interacts with the membrane.^50^ We define the ‘wrapping time’ (*T*_W_) for a NP as the time taken for the wrapping fraction to attain a value of one. The wrapping fraction was defined in our case as the total ligand–receptor binding energy normalized by its potential maximum value, attained when all ligands are bound. Once a vesicle is formed around the nanoparticle, the total number of ligand–receptor bonds remains constant, barring some fluctuations. While not exactly representative of the amount of the surface in contact with the bilayer, it accurately describes the formation of bonds, which leads to wrapping and determines when the NP is fully internalized. We chose this definition rather than trying to estimate the contact between the surface of the membrane and the surface of the nanoparticle because, in our case, unlike for spherical nanoparticles, a completely wrapped particle can still actually have most of its surface without any contact with the membrane due to the presence of the nanostar tips.

We further characterized the impact of shape on the wrapping speed by studying the movement of lipids around the NP. Lipid displacement (|*x̂*|) was measured by determining which lipid heads are involved in wrapping and averaging their displacement every 100 τ, as shown in eq 6, where *x*(τ) is the coordinate of the lipid head at time τ.
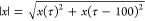
6

On a cell surface, the diffusion of receptors to the particle may increase the uptake time. Therefore, lipid displacement can indicate how lipid mobility is impacted by NP shape.

## Results and Discussion

### Tuning MSiNS Morphology and Size to Cross the BBB

Synthetic factors, such as cosolvent, temperature, surfactant concentration, aging time, and stirring rate, were explored in relation to their effect on the MSiNS diameter and spike length. Shilo et al. reported that nanoparticles with a diameter of 70 nm are optimal to achieve a high accumulation in the brain and so were used here to compare the effects of the diameter on BBB crossing.^51^ An oil/water biphase reaction system was used in the synthesis process. An oil phase (cosolvent) was used to dissolve and dilute the silica source (TEOS) to allow for a more gradual silica growth process, as silica diffuses across the organic phase to the oil/water interphase where the hydrolysis and condensation occur. Given that the temperature influences the time required to transfer sol to gel and consequently the size of nanoparticles, the effect of both cosolvent type and temperature on MSiNS size and spike length were evaluated while keeping the stirring rate constant at 250 rpm, the CTAB concentration at 0.06 M, and aging time at 72 h. Wang et al. previously compared the use of different organic solvents as the oil phase and showed that addition of cyclohexane produces particles with longer spikes than addition of decahydronaphthalene or 1-octadecene.^21^

**Figure 2:**
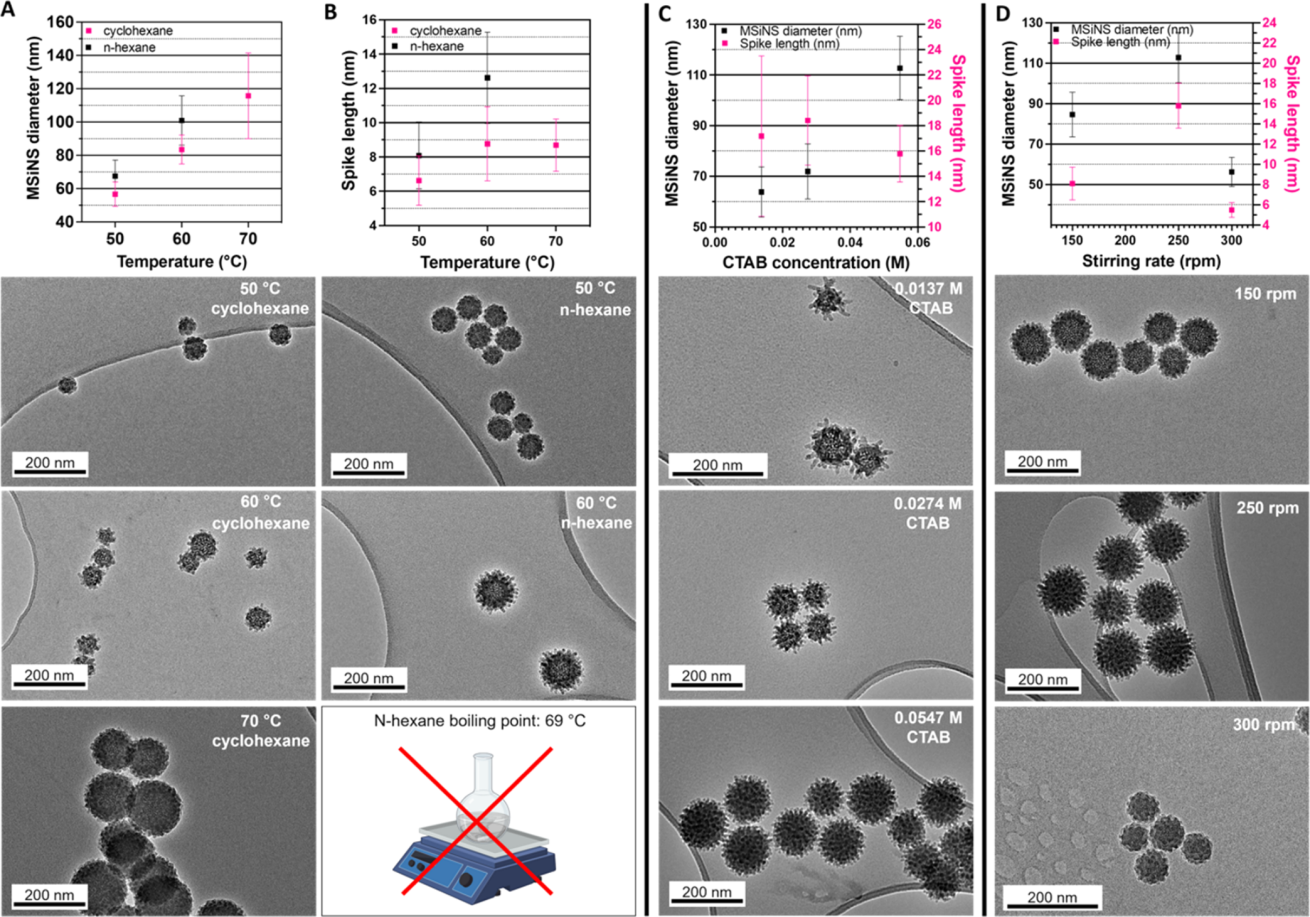
Effect of synthesis parameters on the MSiNS diameter and spike length: Column (A): Effect of the temperature and cosolvent choice on the diameter of nanoparticles; below are TEM images of the as-synthesized nanostructures. Column (B): Effect of the temperature and cosolvent choice on the spike length of MSiNS; below are TEM images of the as-synthesized nanostructures. Column (C): Surfactant (CTAB) concentration effects on the MSiNS diameter and spike length; below are TEM images of the as-synthesized nanostructures. Column (D): Influence of the stirring rate on the MSiNS diameter and spike length; below are TEM images of the as-synthesized nanostructures.

Uniform virus-like MSiNS with an inner spherical mesoporous structure and mesoporous spikes emanating from their core were successfully synthesized following a modified sol–gel single-micelle epitaxial growth process (Figure 2).^21^ The overall spike length, as well as the particle size, increased by substituting cyclohexane (pink squares) with the noncyclical *n*-hexane (black squares) (Figure 2a,b and Table S1). The effect was particularly marked for the MSiNS synthesized in *n*-hexane at 60 °C with the spike length increasing from 9 ± 2 to 13 ± 3 nm. This effect may occur due to the different solubilities of TEOS in the two solvents. *N*-hexane is more polar due to the long carbon chain’s ability to induce dipole–dipole interactions and is less soluble in water.^52^ This means that TEOS is better solubilized in *n*-hexane, and together with the lower miscibility, TEOS is released into the water/cosolvent interface in a more controlled manner. The difference in dynamic viscosity (η) between the two solvents with cyclohexane η = 1 cP and *n*-hexane η = 0.31 cP affected the diffusion coefficients of Si reactive species within the solution and further the hydrolysis rate;^53^ the lower the η of the solvent, the higher the hydrolysis rate, with larger-diameter MSiNS and a longer spike length.

Different synthesis temperatures (50, 60, and 70 °C) were also compared during the synthesis process: (i) to help solubilize CTAB in the water phase and (ii) to increase the rate of silica hydrolysis and condensation. Particles synthesized at 60 °C in cyclohexane had a diameter of 84 ± 9 nm with 9 ± 2 nm spikes; however, when the temperature was increased to 70 °C, the resulting particles were larger (116 ± 26 nm), but the spike length had not changed (9 ± 2 nm). Lowering the temperature to 50 °C resulted in particles with a diameter of 60 ± 7 nm and a spike length of 6 ± 1 nm that appeared to have stopped growing after 72 h. It is likely that the reagents had become depleted, so a temperature of 50 °C may not be high enough to facilitate sufficient diffusion of TEOS into the oil/water interface; therefore, growth became increasingly slow.^54^ The same temperature effect on MSiNS size and spike length was observed when using *n*-hexane with MSiNS growing from 68 ± 10 to 101 ± 15 nm in diameter and from 8 ± 2 to 13 ± 3 nm in spike length as the temperature was increased from 50 to 60 °C. Synthesis using *n*-hexane could not be performed at 70 °C due to the boiling point of the solvent at *T* = 68.7 °C. For this reason, the cosolvent was substituted with *n*-hexane, and a temperature of 60 °C was used to create longer-spiked MSiNS to test on the BBB model.

Next, a study of how reaction time impacts the spike length was performed. A longer reaction time resulted in larger particles and longer spikes (Figure S1). A reaction time of 96 h resulted in a spike length of 16 ± 2 nm, selected as the fixed parameter. A 3D reconstructed electron tomography reconstruction (Figure S2) shows a detailed 3D mesoporous structure of MSiNS with longer spikes.

The critical agent in the MSiNS synthesis is the oil@CTAB+@silica single-micelle formation, which affects formation of the spikes and the mesoporous core. For this reason, we investigated how surfactant concentration affects the MSiNS features. The critical micelle concentration (CMC) of CTAB in water is 0.0009 M.^55^ Above this CMC, CTAB micelles are formed; therefore, three surfactant concentrations, 0.01, 0.03, and 0.06 M, were investigated. The MSiNS diameter increased in proportion to the CTAB concentration, which almost doubled by increasing the concentration 4-fold (Figure 2c). This was in line with the literature that reported that the CTAB plays an important role in the formation of agglomerates by changing the TEOS hydrolysis.^56^ The lower starting CTAB concentration led to an increased ratio of alkoxide hydrolysis with a smaller MSiNS diameter. The length of the spikes did not depend significantly on the initial surfactant concentrations (Table S1). Although the initial surfactant concentration did affect the formation of the tubules on the surface of the mesoporous silica core that initiate spike formation. Generally, the MSiNS grew in three stages: first, the spherical core was formed; then, as the micelle source concentration decreased, some pores on the surface became clogged by partially deposited silica oligomers on which perpendicular single-micelle nanotubes continued growing, with the rest of the template forming the spikes on the core of structured mesoporous silica. Therefore, there appears to be a concentration of the surfactant and silica at which the switch between the growth of the core and generation of spikes occurs. This means that at higher CTAB starting concentrations, there are more micelles for core growth, while at lower concentrations, the “switch” occurs sooner before the core is allowed to grow very large. Consequently, the remaining silica and surfactant are used up for spike growth, but as the concentrations at which the switch happens seemingly depend little on the starting concentration, the mean spike length is similar between samples (from 16 nm with 0.06 M CTAB to 17 nm with 0.01 M CTAB). The standard deviation of the length of the spikes is different between the samples, and this corresponds to a physical difference. The particles synthesized using 0.01 M CTAB had a few long, as well as many short spikes, compared to the 0.06 and 0.03 M CTAB samples, for which the spikes appeared to be much more uniform (Figure 2c).

The stirring rate is another parameter that affects the shape and size of mesoporous silica particles due to production of shear forces during the synthesis process. Different shear forces influence the aggregation of primary particles and silica condensation into a network.^57^ For this reason, the effect of the stirring rate (150, 250, and 300 rpm) on the spike length and MSiNS size was investigated using *n*-hexane as a cosolvent, 0.06 M CTAB, and a 96 h reaction time. The MSiNS diameter and spike length increased from 86 ± 11 to 113 ± 13 nm and from 8 ± 1 to 16 ± 2 nm, respectively, when the stirring rate was increased to 250 rpm, while at the values higher than 250 rpm, a rapidly decreasing value for both features was observed (Figure 2d). This finding was also reported for particle size by Lv et al.^58^ The stirring rate affected the diffusion rate of the TEOS from the oil droplet to the water phase where the hydrolysis occurred. Hence, at a lower stirring rate, the diffusion rate of TEOS from *n*-hexane to water is low, and the concentration of silica monomers at the water/cosolvent interface is much higher than in the inner solution (hexane), leading to larger MSiNS, while increasing the stirring rate accelerates the diffusion of silica monomers with a decrease in local TEOS concentration at the water/cosolvent interface and consequently, smaller MSiNS are obtained. Since a stirring rate of 250 rpm produced the longest spikes, this parameter was fixed for the subsequent syntheses. Figure S3 shows the size and surface charge parameters of the three types of NP tested in the *in vitro* BBB model.

### Immune Effects of MSiNS

After administration of the MSiNS *in vivo*, they can be identified by the immune system as foreign objects and induce immunomodulatory effects. MSiNS can trigger both the adaptive and innate immune systems through nonspecific and specific mechanisms.^59,60^ Some reports indicate that the immune effects of silica nanoparticles vary with their physicochemical properties, such as chemical composition, crystallinity, size, shape, and surface area.^61^ PBMC, together with neutrophils, are the major population of cells involved in an immune reaction; therefore, we evaluated the cytotoxicity and the immunogenic effects of MSiNS in PBMC, and assessed the expression of proinflammatory and anti-inflammatory cytokines.

**Figure 3:**
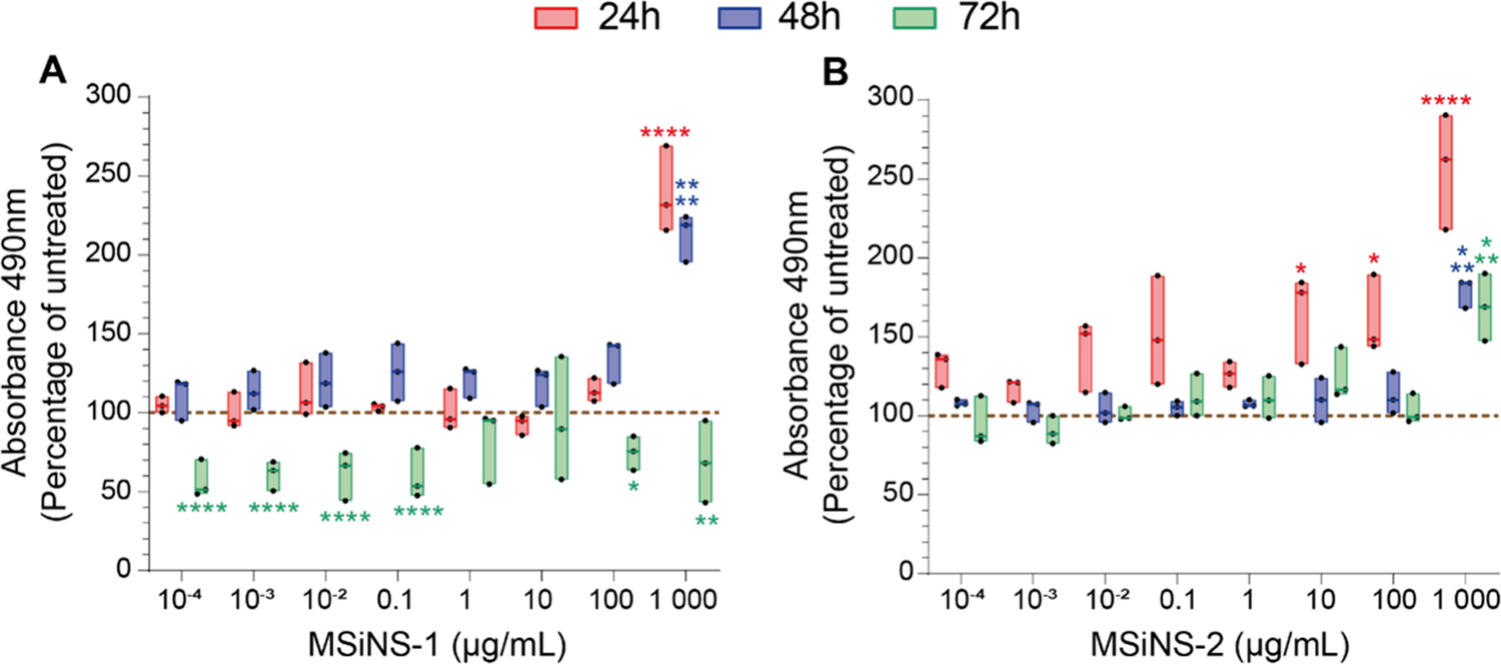
Metabolic activity of PBMC, derived from 3 healthy donors, was assessed using an MTS assay following 24 h (red), 48 h (blue), or 72 h (green) of incubation with various concentrations of (A) MSiNS-1 or (B) MSiNS-2. Results are presented in the percentage of the untreated condition (100%, brown dashed line). The median and minimum/maximum values of 3 different donors are represented in floating bars. Asterisks denote statistically significant data, as defined by two-way analysis of variance (ANOVA) with corrections for multiple comparisons (Dunnett) (*P < 0.05, **P < 0.01, ***P < 0.001, ****P < 0.0001). A 70% viability with respect to the control is considered the limit of toxicity.

NAD(P)H-dependent cellular metabolic activity was measured using the MTS assay in PBMC derived from 3 different healthy donors. There was no significant effect on PBMC viability treated with either MSiNS-1 and MSiNS-2 below (1000 μg mL^–1^) after 24 h (red bar) and 48 h (blue bar) (Figure 3a,b). In contrast, MSiNS-1 (Figure 3a, green bar) were significantly toxic compared to the control after 72 h exposure at all concentrations. This may be due to the shorter spikes of MSiNS-1 that may increase the residence time of MSiNS-1 within the endosomes and promote their maturation inside the lysosomes.^62^ The accumulation of NP inside lysosomes (Figure S4) is known to promote leakage of enzymes that trigger proinflammatory cytokine production.^63^ Moreover, the MTS assay is based on the measurement of NADPH cellular oxidoreductase enzyme activity in the mitochondria, which are very sensitive to NP exposure. The functional interaction between lysosomes and mitochondria is the major cause of oxidative stress and cell death.^64^

**Figure 4:**
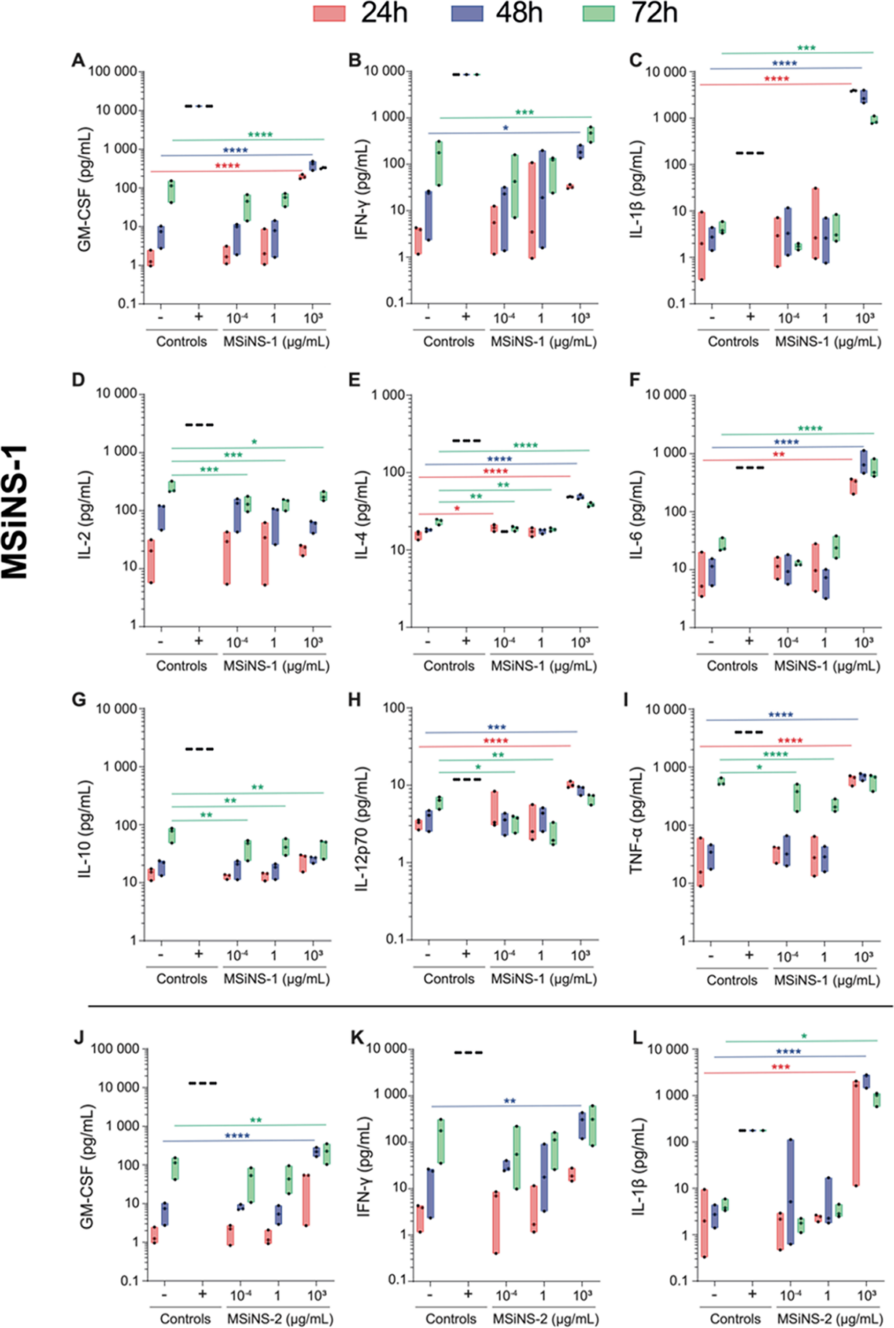

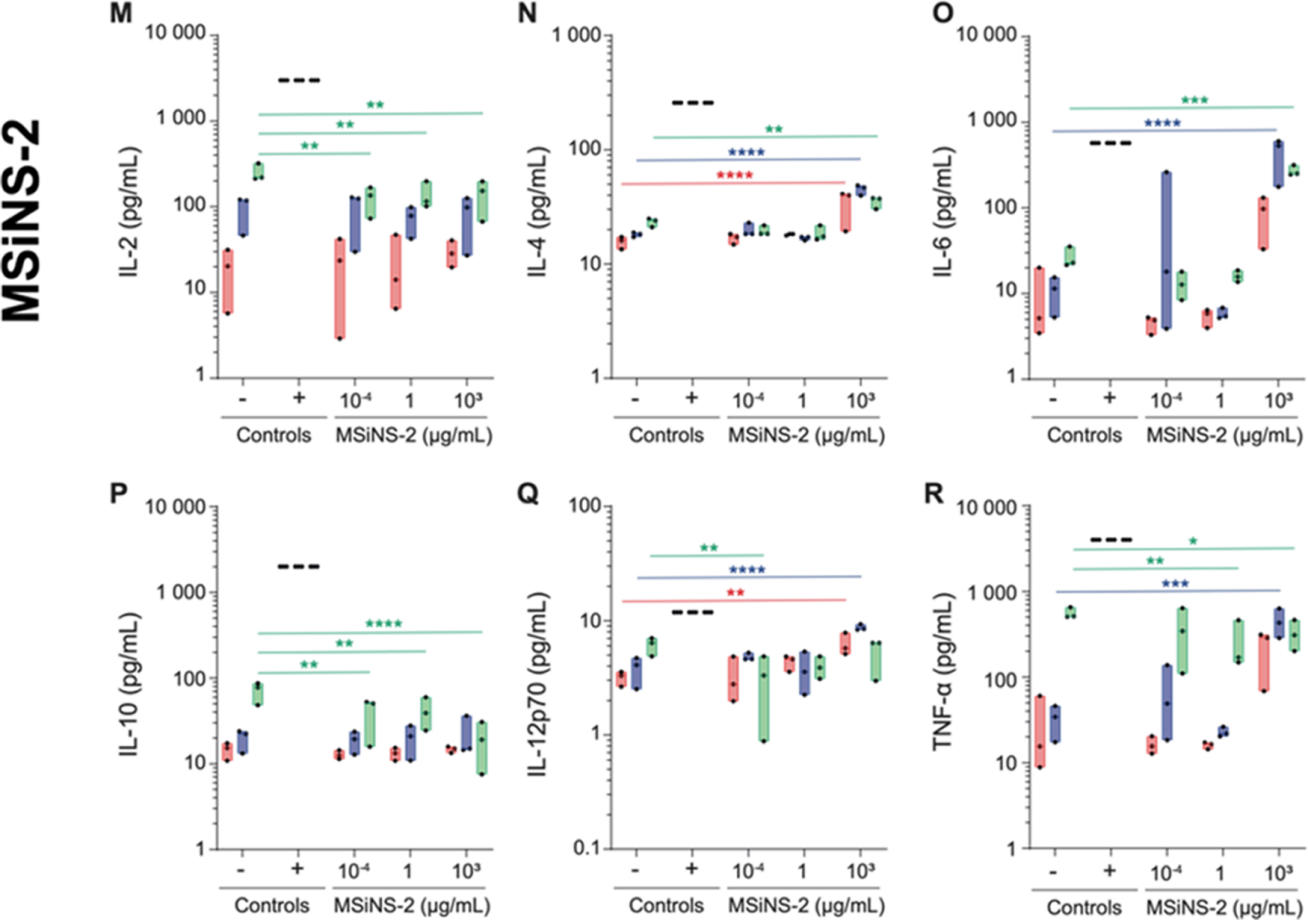
Effect of MSiNS-1 and MSiNS-2 on the expression of proinflammatory and anti-inflammatory cytokines in PBMC cells derived from 3 healthy donors. PBMCs were either left untreated (control −) or incubated for 24 h (red), 48 h (blue), or 72 h (green) with 1 μg/mL Staphylococcal enterotoxin B **+** (control +) or 10^–4^, 1, or 10^3^ μg mL^–1^ (A–I) MSiNS-1 or (J–R) MSiNS-2. Cell-free supernatants were collected to measure (A, J) GM-CSF, (B, K) IFN-γ, (C, L) IL-1β, (D, M) IL-2, (E, N) IL-4, (F, O) IL-6, (G, P) IL-10, (H, Q) IL-12p70, and (I, R) TNF-α production by Luminex. Results are presented as raw data (*i.e*., pg mL^–1^). The median and minimum/maximum values of 3 different donors are represented in floating bars. Asterisks denote statistically significant data as defined by two-way analysis of variance (ANOVA) with corrections for multiple comparisons (Dunnett) (*P < 0.05, **P < 0.01, ***P < 0.001, ****P < 0.0001).

The length of MSiNS influences the cytokine secretion and how the selection of NP shape could prevent a strong immune response and allow for more efficient drug delivery (Figure 4). Both types of MSiNS did not have a significant effect on the production of GM-CSF (a, j), IFN-γ (b, k), IL-2 (d, m), IL-12p70 (h, q), TNFα (i, r) and proinflammatory cytokines at all concentrations and until 72 h compared to the cells not treated with MSiNS and to the SEB positive control. SEB is a toxin with a highly toxic effect on the immune system that stimulates cytokine release and inflammation.^65^ Conversely, both types of MSiNS at the highest concentration caused a minor increase in anti-inflammatory IL-4 (e, n). GM-CSF, IFN-γ, and IL-4 are associated with macrophage polarization. GM-CSF and IFN-γ, a Th1 cytokine, promote macrophage polarization toward the M1 (or inflammatory) form, while IL-4, a Th2 cytokine, promotes their polarization toward the M2 (or anti-inflammatory) form.^66^ Qie et al. reported that the degree of NP internalization is significantly higher in M1 macrophages than M2 macrophages.^67^ Thus, MSiNS seem to have a lower probability of being taken up by macrophages, preventing extensive metabolism and prolonging the NP circulation time. Our MSiNS had no significant effect on IL-12p70 and IL-2, which are considered a bridge between adaptive and innate immunity involved in T-cell activation.^68,69^ A fundamental understanding of the body’s reaction to MSiNS exposure is of crucial importance as this may impact their biocompatibility and safety.^70^ An acute inflammation with the upregulation of TNF is reported to be involved in the first body reaction to foreign objects.^71^ Hence, we have quantified TNF cytokine in PBMC cells treated with MSiNS; no significant change was detected up to 72 h and at the highest concentration (10^3^ μg mL^–1^) (Figure 4i,r). This implies that the MSiNS are biocompatible and do not produce significant acute inflammation. In contrast, the highest concentration (10^3^ μg mL^–1^) of both MSiNS-1 and MSiNS-2 upregulated the proinflammatory IL-6 (f, o) and IL-1β (c, l) cytokines. Based on these results and on the fact that IL-6 and IL-1β can have a synergistic effect on neurotransmitter alterations,^72^ we excluded the use of the (10^3^ μg mL^–1^) MSiNS concentration for testing on the BBB *in vitro* model.

### Neurological Toxicity of MSiNS

**Figure 5:**
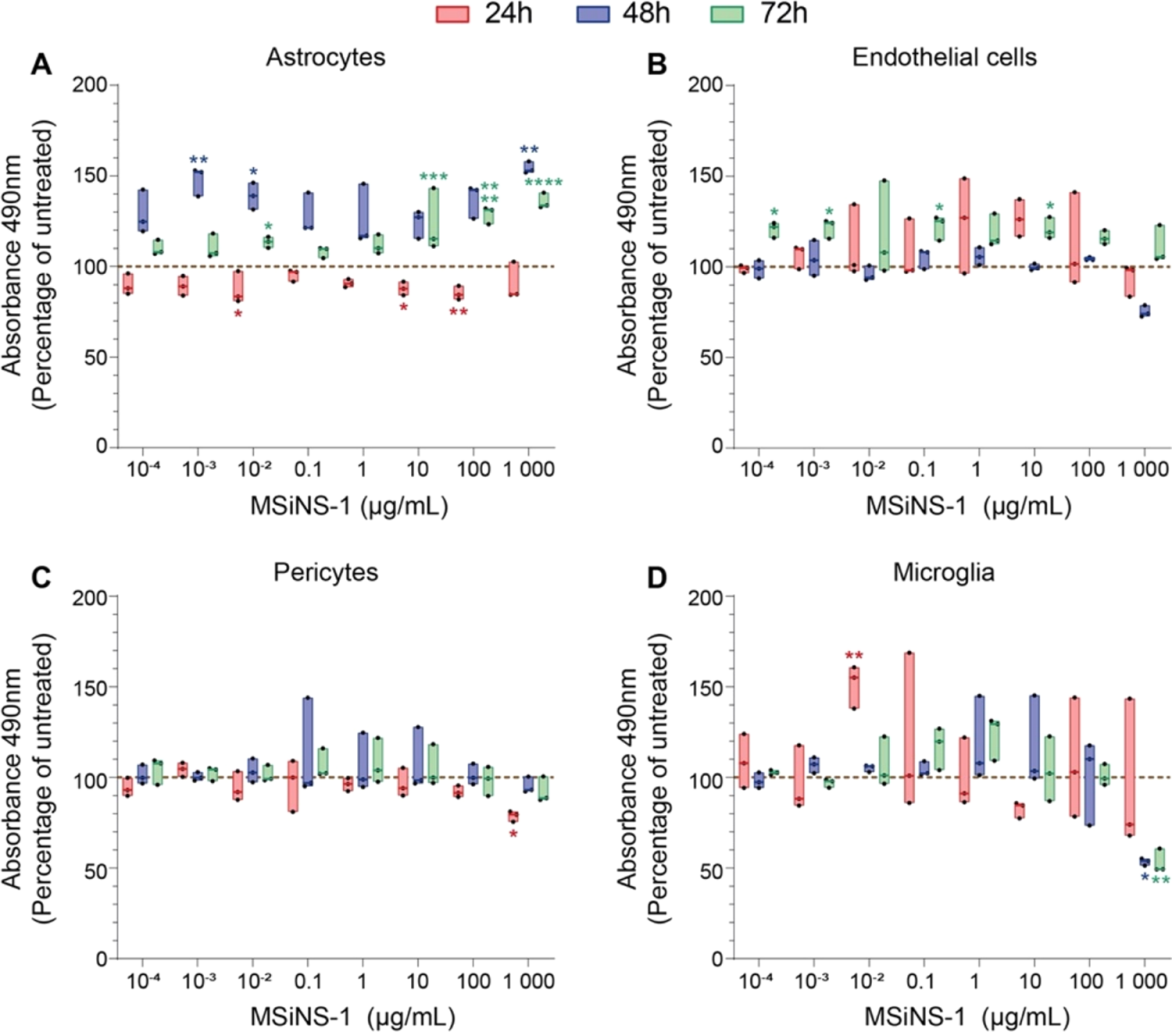
Metabolic activity of (A) astrocytes, (B) endothelial (hCMEC/d3), (C) pericytes (HBVP), and (D) microglia (HMC3) cells assessed using an MTS assay, following 24 h (red), 48 h (blue), or 72 h (green) of incubation with various concentrations of MSiNS-1. Results are presented as a percentage of the untreated condition (100%, brown dashed line). The median and minimum/maximum values of one experiment in triplicate are represented in floating bars. Asterisks denote statistically significant data as defined by two-way analysis of variance (ANOVA) with corrections for multiple comparisons (Dunnett) (*P < 0.05, **P < 0.01, ***P < 0.001, ****P < 0.0001). A 70% viability with respect to the control is considered the limit of toxicity.

Before setting up a BBB *in vitro* model, the cytotoxicity of MSiNS-1 was assessed singly in all types of neural cells (endothelial, astrocytes, pericytes and microglia) that make up the multicellular culture. An MTS assay was used to measure the cellular metabolic activity before and after MSiNS-1 treatment at different time points (24, 48, 72 h). There were no significant changes in the viability of any type of the neural cells treated with MSiNS-1 up to a concentration of 100 μg mL ^–1^ after 24 (red bar) and 48 h (blue bar) (Figure 5a–d), while MSiNS-1 were significantly toxic for microglia at 48 (blue bar) and 72 (green bar) h exposure at concentrations above 100 μg mL^–1^ (*P* < 0.05 and *P* < 0.01) (Figure 5d). Considering the viability results from PBMC and neural cells, the safest (1 μg mL ^–1^) concentration to test the MSiNS and MSiNP on the BBB cells was selected.

### MSiNS’ Capacity to Translocate the BBB

**Figure 6:**
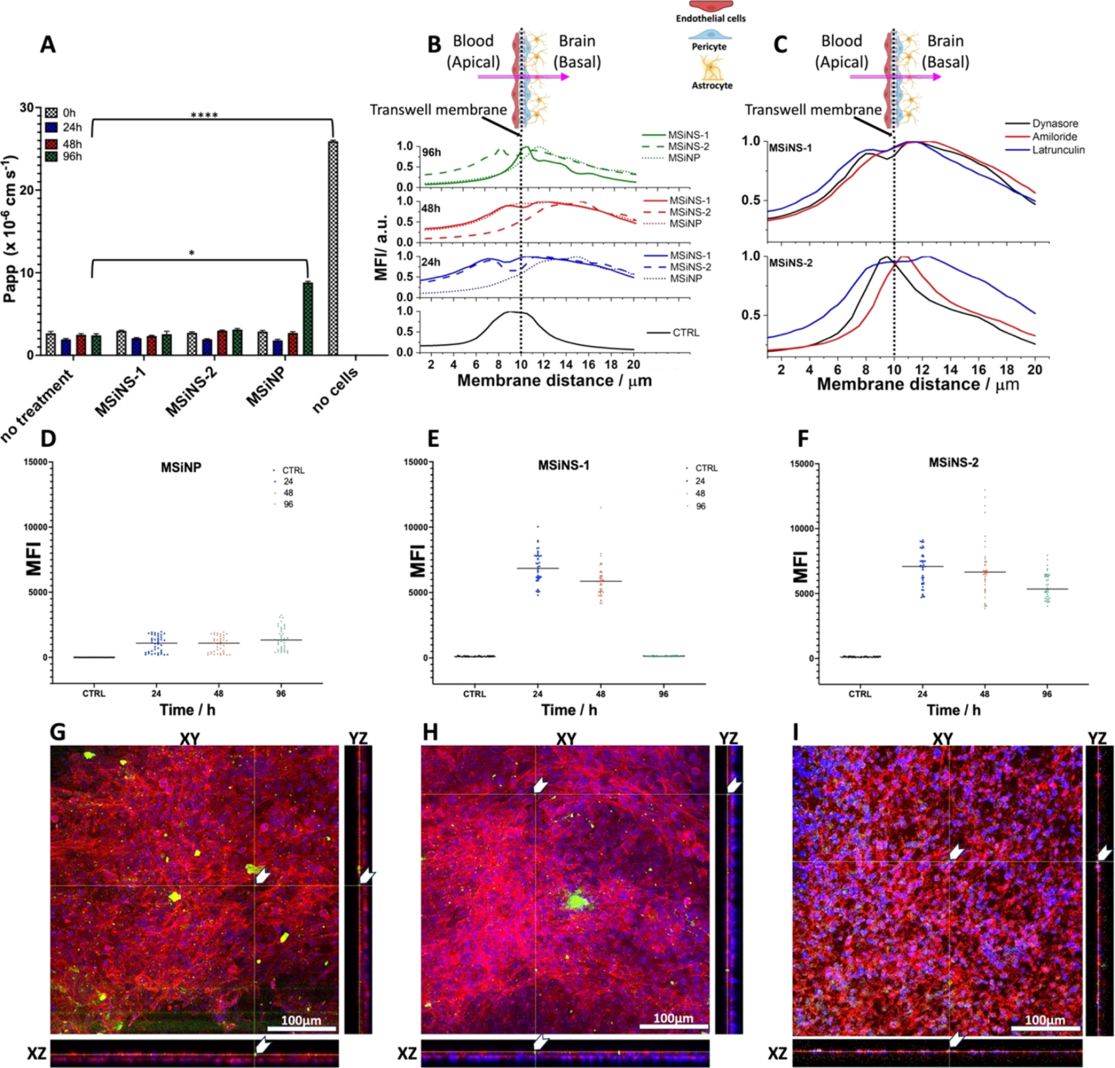
BBB permeability and translocation of short (MSiNS-1) and long (MSiNS-2)-spiked and MSiNP. (A) BBB permeability coefficients calculated from the diffusion of 70 kDa dextran–rhodamine (0.5 mg mL^–1^) through a membrane (no cells) and a coculture/multicellular transwell BBB model with and without MSiNS and MSiNP treatment (1 μg mL^–1^) at 0 (black bar), 24 (blue bar), 48 (red bar), and 96 h (green bar). Asterisks denote statistically significant data, as defined by two-way analysis of variance (ANOVA) with corrections for multiple comparisons (Dunnett) (*P < 0.05, ****P < 0.0001). (B) NP position with respect to the inset membrane (membrane distance) after 24, 48, and 96 h. (C) NP position with respect to the inset membrane after using the molecular trans-endothelial transport inhibitors latrunculin A (blue line), dynasore (black line), and amiloride (red line) to inhibit actin polymerization, dynamin GTPase activity, and micropinocytosis, respectively. Mean fluorescence intensity (MFI) and median (black line) of (D) MSiNP, (E) MSiNS-1, and (F) MSiNS-2 after 24, 48, and 96 h. Side projections (xy, xz) of confocal Z-stack images, confirming the presence of (g) MSiNP, (h) MSiNS-1, and (i) MSiNS-2 (green) on the basolateral side of the *in vitro* BBB model.

A coculture/multicellular model allowing for cell-to-cell contact^26,73^ was used for testing the MSiNS/MSiNP capacity to cross the BBB. This comprises four human-derived cell types, endothelial cells on the apical side, a mixed culture of astrocytes and pericytes on the basolateral side of the porous insert, and microglia cells on the bottom of the culture plate, used for testing MSiNS/MSiNP crossing. The BBB integrity was evaluated before and after MSiNS/MSiNP treatment (1 μg mL^–1^) by measuring its permeability to dextran–rhodamine B (70 kDa) (Figure S5) and calculating the BBB apparent permeability coefficient (*P*_app_ (cm s^–1^)) (Figure 6a; eq 7) after 0 (black bar), 24 (blue bar), 48 (red bar), and 96 (green bar) h.
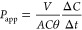
7where *V* is the basolateral volume chamber (750 μL), *A* is the surface area of the inset membrane (0.3 cm^2^), *C*θ is the initial concentration in the apical chamber (0.5 mg mL ^–1^), Δ*C* is the diffusion concentration of the basolateral chamber, and Δ*t* is the diffusion time (4 h). A control insert with no cells was used to measure the passive diffusion of the dextran–rhodamine B through a BBB with a 100% permeability.

The BBB model maintained the barrier function for at least 4 additional days (no treatment), which enabled time–course studies of the NP crossing and their effect on BBB integrity (Figure 6a). None of the MSiNS produced any significant reductions in the permeability until at 48 h, while the MSiNP increased the passage of the dextran–rhodamine B with the permeability increasing significantly up to 50% with respect to the untreated control only after 96 h (Figure S5). The MSiNP may be accumulated preferentially in lysosomes, promoting proinflammatory cytokine production. Different studies have also demonstrated, *in vitro* and *in vivo*, that immunological, chemical, or physical^74,75^ insults are responsible for the increase in BBB permeability and strictly correlated with the release of proinflammatory cytokines. Trickler et al.^76^ showed that specific NP compositions and sizes can cause a significant proinflammatory response that can influence the integrity of the BBB with proinflammatory cytokine upregulated by Cu and Ag NPs but not by Au. Liu et al.^77^ have demonstrated, *in vitro* and *in vivo*, that 20 nm MSiNP could disturb the BBB structure and induce inflammation; nonetheless, to the best of our knowledge, no one has shown the effect of MSiNS on BBB permeability.

To mimic the MSiNS crossing from blood circulation into the brain, MSiNS (1 μg mL^–1^), previously conjugated with florescent dye (FITC, green), were added to the apical side of the BBB and imaged in 3D using confocal microscopy. Images were acquired at 24, 48, and 96 h post-treatment from the apical to the basal side of the multicell layers (≈10 μm). The mean fluorescence intensity (MFI) of the MSiNS and MSiNP at different z-stack heights with respect to the transwell membrane (black dashed line) is shown in Figure 6b. At all three time points, a general trend of MSiNS and MSiNP crossing was measured by analyzing the shift in the fluoresce intensity with respect to the transwell membrane. The highest translocation rate was for MSiNS-1 with short spikes, indicated by a decrease in MFI to zero at the basolateral side. The crossing of MSiNS-1 was confirmed by the green fluorescence detected in the microglia cells at the bottom of the well after 96 h (Figure S6). Although the MSiNP more efficiently crossed the multicell layers of the BBB model within the first 24 h, after 96 h, they seem to become entrapped in the basolateral compartment formed by an astrocyte and pericyte mixture. On the contrary, the MSiNS-2 with longer spikes translocated the BBB more rapidly in the first 48 h but with the intensity increased after 96 h in the apical side, implying that they had accumulated in this region.

The calculated median of the MFI of the three NP through the 3 layers of cells that constituted the BBB is shown in Figure 6d–f. MSiNS-1 translocated across the BBB model more rapidly than MSiNP and MSiNS-2 that accumulate in the basolateral and apical side, respectively. Confocal microscopy of all NP types indicated that they were present in the basolateral compartment formed by astrocyte and pericyte coculture (Figure 6g–i).

To better understand the underlying endocytosis molecular pathways that mediated the trans-BBB transport of MSiNS, the involvement of the major molecular pathways in trans-endothelial transport was evaluated, including phagocytosis, clathrin-mediated endocytosis, and micropinocytosis (Figure 6c). Latrunculin A (blue line), dynasore (black line), and amiloride (red line) were used to inhibit actin polymerization, dynamin GTPase activity, and micropinocytosis, respectively.^78^

Figure 6c suggests that cellular uptake is the primary mechanism of BBB penetration rather than movement through tight junctions. Translocation of MSiNS-1 across the BBB was not affected by any of the inhibitors, while MSiNS-2 was significantly impacted by dynasore and amiloride, implying that cellular uptake of MSiNS-2 may be mediated *via* clathrin-mediated endocytosis and macropinocytosis, respectively. On the other hand, the fact that there was no change in distribution when latrunculine was added shows that the actin cytoskeleton is not a major contributor to entry or migration of the nanoparticles, and MSiNS-1 follow preferentially the phagocytosis.

The difference between MSiNS-1 and −2, which share an equivalent total radius of 50.2 nm, in BBB penetration can be related to their tip size and density. Here, these are characterized together as solidity, which is the particle’s projection area divided by the area of its convex hull. These values were measured using the TEM images of 30 MSiNS from each synthesis method in OpenCV. The solidity of MSiNS-1 and MSiNS-2 is 0.87 and 0.79, respectively, implying that the increased tip length of MSiNS-2 results in a lower density of tips. Since the total radius of both particles is the same, a longer tip length would imply a smaller core radius with a smaller surface area to support tips.

### Modeling MSiNS/MSiNP Cell Interactions

Molecular dynamics was used to determine the influence of MSiNS geometry on cellular uptake speed. A coarse-grained simulation was used, as described above, which allowed us to study MSiNS/MSiNP for a long enough time to observe their uptake by the cell membrane. The average lipid bilayer thickness was determined to be 4.51 ± 0.04 σ, where σ is the fundamental unit of distance.^79^ The thickness is in good agreement when compared with experimentally derived measurements of lipid bilayer thicknesses, which are around 5 nm.^80^ With this approximation, we estimated that σ = 1 nm.

In our simulations, NP endocytosis is driven by ligands on the surface of the NP, binding to receptors in the lipid bilayer. The formation of these bonds counteracts the deformation energy required to bend the lipid bilayer around the NP.^45,81,82^ We note that during the wrapping process, a lipid bilayer can explore new areas of the NP surface through thermal fluctuations: the natural undulations in the bilayer. In the absence of empirical data regarding patch size, shape, or distribution, we assumed that ligands form uniformly distributed patches on the MSiNP surface. We explored multiple patch sizes that would approximate the limits of the thermal fluctuations within our system. While the validity of this assumption is not explored experimentally within this manuscript, it gives us more control over the study. The uptake rate of 7 MSiNP, each with different ligand patch sizes, was compared to MSiNS with 35 tips (Table 2). Each NP had the same total number of ligands on the surface. Thus, the total maximum ligand–receptor energy is the same for all NPs, and the only difference between them is due to their geometry (spherical *vs* star) and the location of ligands.

**Table 2:** Relative Time to Uptake MSiNP with Different Ligand Distributions, Compared to MSiNS of the Same Maximum Radius with 35 Tips^a^

patches	ligands/patch	sep. dist (σ)	*T*_w-MSiNP_/*T*_w-MSiNS_
56	5	2.8	0.8
40	7	3.2	1.0
35	8	3.5	1.1
28	10	3.8	1.6
20	14	4.4	1.7
14	20	5.1	N/A
10	28	5.6	N/A

a N/A indicates that the MSiNP was not fully taken up by the lipid bilayer membrane.

We clearly observed that the average separation distance between the center of the patches on the spherical nanoparticle, MSiNP, influenced the wrapping time *T*_w_. A smaller distance between patches means that smaller lipid bilayer fluctuations are sufficient to contact the next ligand. When a critical separation distance is reached, larger thermal fluctuations become necessary to access new patches, which is energetically unfavorable. Interestingly, we also noticed that MSiNS with 35 tips were endocytosed faster than the equivalent MSiNP.

**Figure 7:**
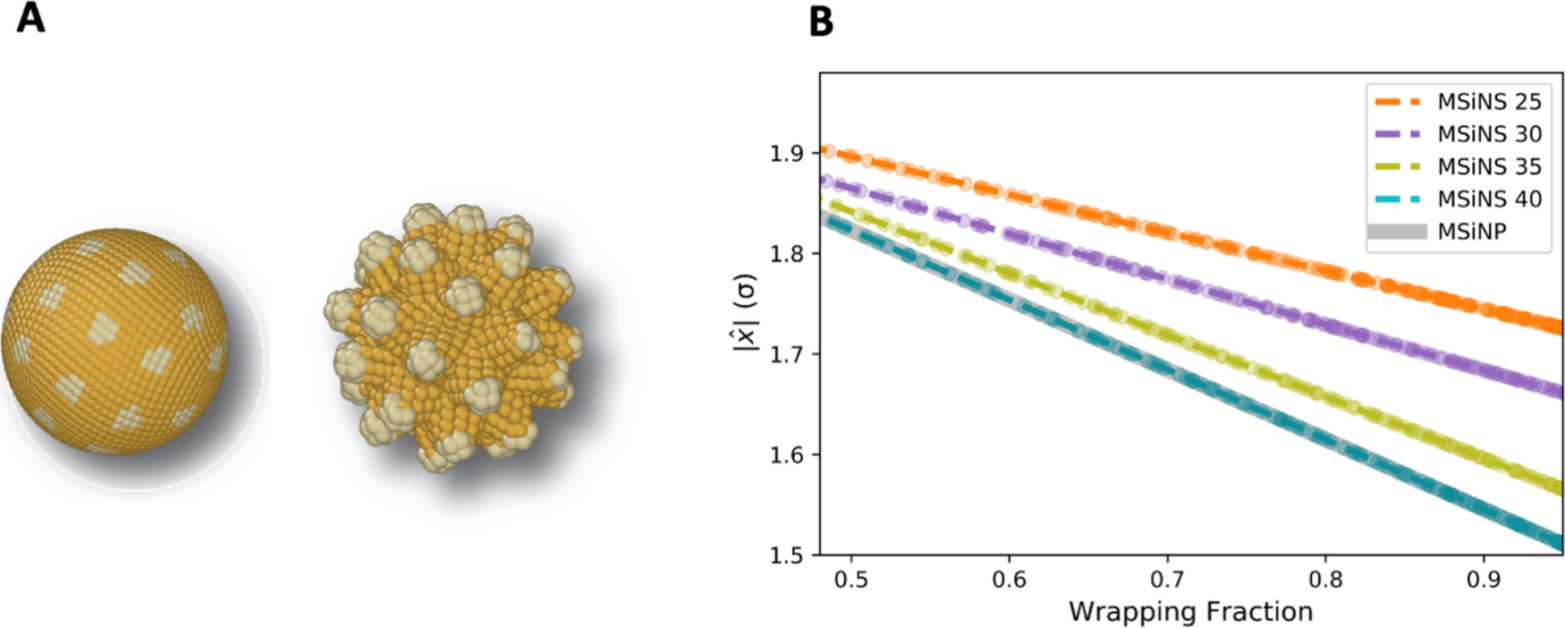
(A) MSiNP (left) and MSiNS (right) with colored ligands to demonstrate placement on the nanoparticle surfaces. (B) As the number of tips increases from 25 to 40, the MSiNS more closely resemble a sphere, such as MSiNP, reducing the displacement of lipids involved in wrapping the particles.

We simulated the interactions between the modeled cell membrane and MSiNS with 10–40 tips to determine if MSiNP were also affected by the increased separation distances between binding spots. Figure 7a shows the final state of the nanostars with 10–25 tips. Similar to what we observed for MSiNP with patches in Table 1, in the case of nanostars, an increase in the average of the tip-to-tip distance appears to limit the ability of the membrane to reach the ligands on the subsequent tips.

The next task was to determine the reason for which MSiNS with 35 tips have a faster uptake time than the comparative MSiNP. To investigate the mechanism behind this enhanced uptake, the displacement of individual lipids, *x̂*|, was measured as a function of particle wrapping. Only the lipids present in the final vesicle were measured to reduce noise in the results. The results shown in Figure 7b suggest that when interacting with MSiNS, the lipids experience a higher mobility compared to when they interact with MSiNP. The intertip spaces act as relief areas, facilitating lipid reorganization during MSiNS wrapping. The membrane’s increased adaptability during MSiNS interaction can facilitate a more efficient wrapping process than for the MSiNP.

The separation distance between MSiNS tips appears to be a crucial determinant of cellular uptake and passage through the BBB. If the separation distance is small enough, the MSiNS modeled here have equivalent or better performance to a spherical nanoparticle (MSiNP). If the tip-to-tip separation distance is too large, then lipid bilayer fluctuations alone may be insufficient to uptake the nanoparticle fully. The reliance of MSiNS-2 on various mechanisms of endocytosis, such as micropinocytosis, implies that the separation distance between tips may be too large, hindering uptake.

### *In Vivo* Mesoporous Silica Nanostar Distribution and Biosafety Evaluation

Our *in vitro* studies have shown that MSiNS translocated across the BBB model, with MSiNS-1 having a higher crossing rate with respect to MSiNP and MSiNS-2. We then tested whether MSiNS-1 was toxic *in vivo* by recording any brain weight changes and histopathology analysis and could accumulate inside the brain by silicon quantification. Healthy mice were treated with an IV 10 mg/kg single dose of MSiNS-1 *via* the lateral tail vein. After 3, 7, and 24 h postdosing, silica was quantified in both blood and brain, and their toxicity was evaluated. Figure S7(a,b) clearly shows a simultaneous decrease of MSiNS-1 concentration in the blood and accumulation of silicon in the brain after 7 h of treatment. The concentration of MSiNS-1 in the brain did not increase significantly up to 24 h. We did not observe any significant brain weight changes (Figure S7c) after MSiNS-1 administration at all time points, compared to the nontreated control mice. Moreover, a detailed microscopic examination of brain tissue (Figure S7d) did not reveal any morphological changes or inflammatory infiltrates.

## Conclusions

A biodegradable, nontoxic NP with virus-like shape that crosses a unique *in vitro* BBB coculture model, composed of four types of neuronal cells, was engineered. MSiNS-1 with shorter spikes (9 ± 2 nm) more rapidly crossed the *in vitro* BBB model than MSiNS-2 with longer spikes (18 ± 4 nm). The MD simulations corroborated the *in vitro* studies showing a more effective endocytosis of MSiNS than MSiNP due to an increase of membrane wrapping toward the spike in MSiNS. A modified sol–gel process was designed to produce uniform and tunable virus-shaped MSiNS/MSiNP by controlling the surfactant concentration, stirring rate, temperature, and type of the cosolvent. The MSiNS showed no cytotoxicity or immunogenicity at concentrations of up to 1 μg mL^–1^ on PBMC and neuronal cells. The *in vitro* BBB model maintained the barrier function up to 96 h, allowing for time–course studies of the NP crossing and their effect on BBB integrity. None of the MSiNS/NP modified the permeability of the BBB until 48 h, while the MSiNP increased the BBB permeability up to 50%, with respect to the untreated control after 96 h.

Cellular uptake was the primary mechanism of BBB penetration rather than movement through the tight junctions. MSiNS-2 with longer spikes were taken up *via* a combination of clathrin-mediated endocytosis and micropinocytosis, while MSiNS-1 preferentially followed the phagocytosis uptake pathway. MD simulations confirmed that, under certain conditions, the cell membrane can more quickly wrap around nanostars than the spherical particles. Moreover, the same simulations showed that the lipids involved in the wrapping process had a higher mobility for the nanostars, possibly due to the looser pinning to the nanoparticle surface. A higher mobility will speed up membrane rearrangement during wrapping, providing a potential explanation for this enhanced uptake. The ability of these MSiNS to cross the BBB *in vitro* and *in vivo*, without producing any structural modifications to the brain tissue, highlights their potential therapeutic value for the treatment of brain diseases and to reduce the use of high drug doses without unwanted side effects.

## Data Availability

The data that support the findings of this study are available from the corresponding authors upon reasonable request.

## Supporting Information Available

Nanostar features tuned by changing synthesis conditions, importance of aging time and solvent type for nanostar size, 3D nanostar TEM image, nanoparticles for *in vitro* studies, fluorescent nanostar uptake into the cells, BBB permeability measurement with and without nanoparticle treatment, nanostar and microglia interaction, simulation method and nanoparticle shape, and nanostar *in vivo* examination (PDF)

## Notes

Human fetal brain tissues from 15 to 20 weeks’ fetuses were obtained from the MRC—Wellcome Trust Human Developmental Biology Resource (HDBR), UCL, with ethical approval (University College London, UCL, site REC reference: 18/LO/0822-IRAS project ID: 244325 and Newcastle site REC reference: 18/NE/0290-IRAS project ID: 250012). All animal studies were reviewed and approved by the Hackensack Meridian Health Institutional Animal Care and Use Committee.
