# Supplementary material for: Virus-Shaped Mesoporous Silica Nanostars to Improve the Transport of Drugs across the Blood–Brain Barrier
Source: ACS Appl Mater Interfaces. 2024 Jul 11;16(29):37623–40. doi: 10.1021/acsami.4c06726 (PMC11284754; doi:10.1021/acsami.4c06726)
Supplement: Supplementary file 1 — am4c06726_si_001.pdf [file am4c06726_si_001.pdf]

“Supporting Information”

VIRUS SHAPED MESOPOROUS SILICA NANOSTARS TO IMPROVE TRANSPORT OF DRUGS ACROSS THE BLOOD–BRAIN BARRIER.

Alessandra Pinna[* γ 1,2,3], Ieva Ragaisyte[γ3], William Morton[3], Stefano Angioletti-Uberti[3], Alizé Proust[2], Rocco D'Antuono[8,10], Chak Hon Luk[2], Maximiliano G. Gutierrez,[2] Maddalena Cerrone,[2] Katalin A. Wilkinson,[2,5,6] Ali A. Mohammed,[4,9] Catriona M. McGilvery,[3] Alejandro Suárez-Bonnet[2,11], Matthew Zimmerman[12], Martin Gengenbacher[12,13], Robert J. Wilkinson,[2,5,6,7], Alexandra E. Porter[3]

[1] School of Veterinary Medicine, Faculty of Health and Medical Sciences, University of Surrey, Guildford GU2 7XH, UK

[2] The Francis Crick Institute, NW1 1AT, London, UK

[3] Department of Materials, Imperial College London, SW7 2AZ, London, UK

[4]Dyson School of Design Engineering, Imperial College London, SW7 2AZ, London, UK

[5]Centre for Infectious Diseases Research in Africa (CIDRI-Africa), Institute of Infectious Disease and Molecular Medicine, University of Cape Town, Observatory 7925, Republic of South Africa

[6]Department of Medicine, University of Cape Town, Observatory 7925, Republic of South Africa

[7]Department of Infectious Diseases, Imperial College London, W12 0NN, UK

[8]Crick Advanced Light Microscopy STP, The Francis Crick Institute, NW1 1AT, London, UK

[9]School of Design, Royal College of Art, SW11 4AY, London, UK

[10] Department of Biomedical Engineering, School of Biological Sciences, University of Reading, Reading, RG6 6AY, UK

[11]Department of Pathobiology and Population Sciences, The Royal Veterinary College, North Mimms, Hatfield, Hertfordshire AL9 7TA, UK

[12]Center for Discovery and Innovation, Hackensack Meridian Health, 111 Ideation Way, Nutley NJ 07110, United States

[13]Hackensack Meridian School of Medicine, Nutley, NJ 07110, United States

[γ] Shared first authorship

[*] Corresponding author; a.pinna@surrey.ac.uk

Table S1. Synthesis conditions, corresponding particle size and spike length of MSiNS samples. N= number of particles or spikes sampled in the Fiji analysis.

Sample name	TEM analysis				Synthesis conditions				
	Particle size (nm)	N	Spike length (nm)	N	CTAB concentration (M)	Aging time (h)	Stirring rate (rpm)	Co-solvent	Temperature (°C)
MSiNS-1	83 ± 9	33	9 ± 2	50	0.06	72	250	Cyclohexane	60
MSiNS-2	72 ± 11	30	18 ± 4	31	0.03	96	250	n-Hexane	60
MSiNS-3	56 ± 12	28	9 ± 2	50	0.03	72	250	Cyclohexane	60
MSiNS-4	55 ± 10	50	8 ± 2	50	0.01	72	250	Cyclohexane	60
MSiNS-5	92 ± 10	100	4 ± 1	50	0.03	96	250	Cyclohexane	60
MSiNS-6	49 ± 5	41	5 ± 1	50	0.03	96	250	Cyclohexane	60
MSiNS-7	116 ± 26	34	9 ± 2	20	0.06	72	250	Cyclohexane	70
MSiNS-8	57 ± 7	62	7 ± 1	50	0.06	72	250	Cyclohexane	50
MSiNS-9	113 ± 12	68	16 ± 2	25	0.06	96	250	n-Hexane	60
MSiNS-10	68 ± 10	20	8 ± 2	23	0.06	72	250	n-Hexane	50
MSiNS-11	68 ± 7	12	10 ± 2	23	0.06	96	250	n-Hexane	50
MSiNS-12	85 ± 11	67	8 ± 2	50	0.06	96	150	n-Hexane	60
MSiNS-13	56 ± 7	92	5 ± 1	50	0.06	96	300	n-Hexane	60
MSiNS-14	64 ± 10	38	17 ± 6	50	0.01	96	250	n-Hexane	60
MSiNS-15	101 ± 15	48	13 ± 3	50	0.06	72	250	n-hexane	60

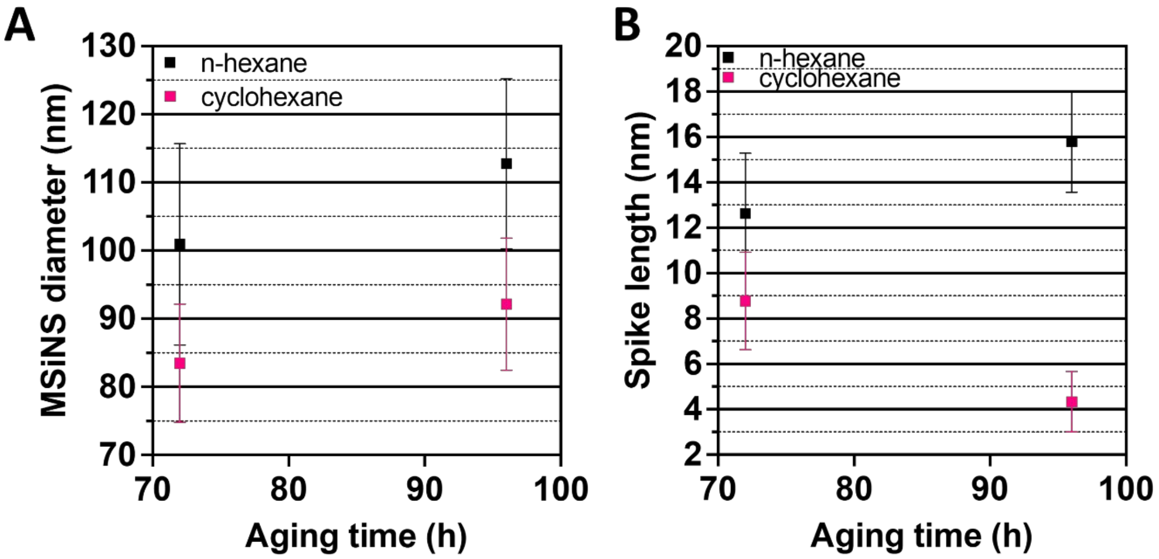

Figure S1. Aging time on MSiNS A) nanoparticle diameter and B) spike length in two oil phase solvents.

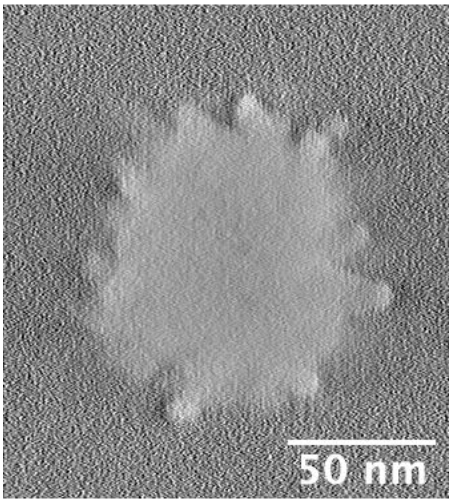

Figure S2. 3D reconstructed electron tomography images of MSiNS-2.

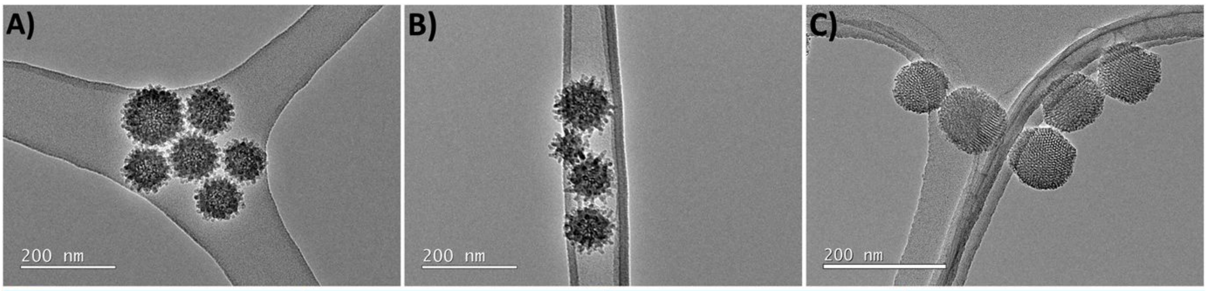

D) Sample name	Z-Average (nm)	PDI	ζ-potential (mv)
MSiNS-1	175.07 (±1.99)	0.244 (±0.008)	-36.13 (±1.27)
MSiNS-2	150.37 (±5.87)	0.217 (±0.039)	-39.10 (±0.44)
MSiNP	90.72 ± (11.91)	0.68 (±0.18)	-31.39 (± 0.42)

Figure S3. TEM images of A) MSiNS-1 (short spike), B) MSiNS-2 (long spike) nanostars and C) MSiNP (spherical). D) MSiNS-1, MSiNS-2 and MSiNPs (10ppm in PB, pH7.2) hydrodynamic diameter (nm), polydispersity index (PDI) and zeta potential (mV). The results are express as mean of 3 replicates and SD.

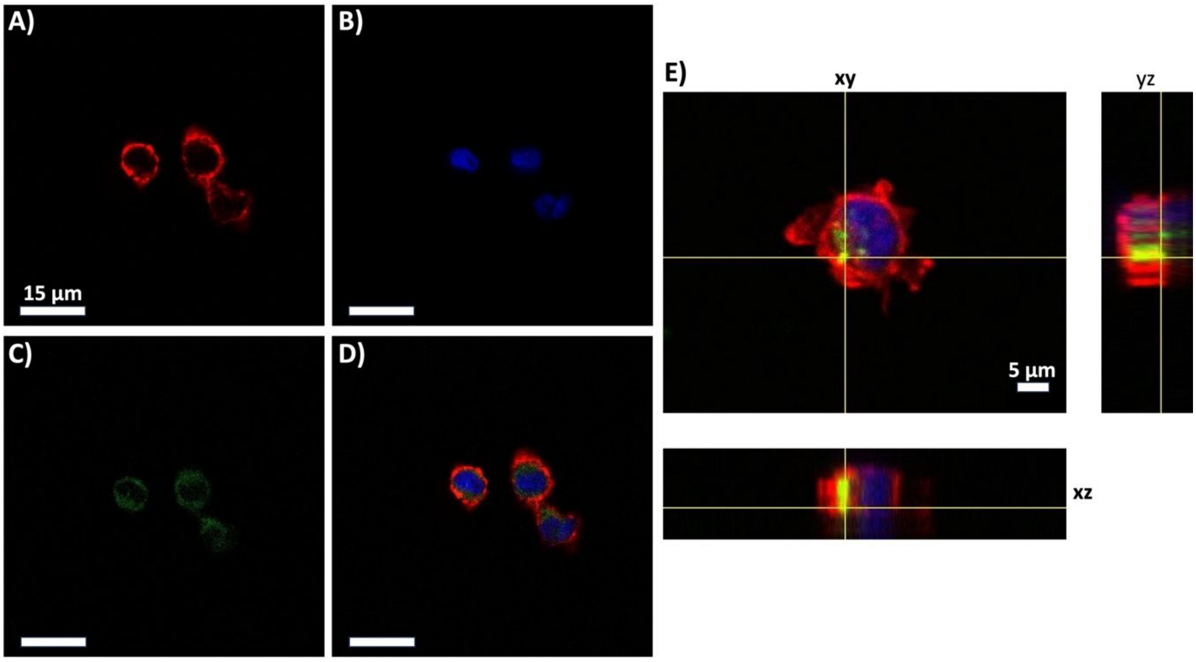

Figure S4. Confocal images of PBMC cells incubated with 1µg mL $^{-1}$ of FITC–MSiNS-1 for 24h. A) Cytopainter F-actin for cytoplasm (red); B) DAPI staining for nucleus (blue); C) FITC for MSNS-1(green) and D) merged images of the 3 channels (Scale bar; 15 µm). E) Side projections of confocal Z-stack confirming MSNS-1 (green spots) internalization in PBMCs (Scale bar; 5 µm).

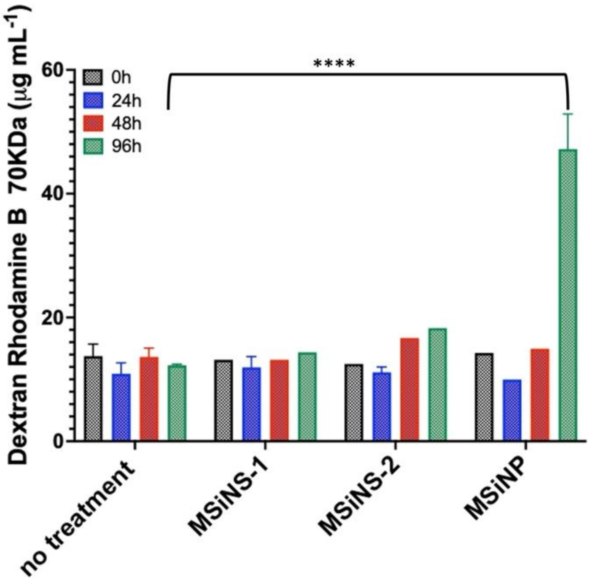

Figure S5. Dextran Rhodamine B (70KDa) concentrations in the basolateral compartment calculated from the diffusion of 70 kDa dextran-rhodamine (0.5 mg mL-1) through a membrane (No cell) and a co-culture /multicellular transwell BBB model with and without MSiNS and MSiNP treatment (1 μg mL-1) at 0 (black bar), 24h (blue bar), 48h (red bar), 96h (green bar). Asterisks denote statistically significant data as defined by two-way analysis of variance (ANOVA) with corrections for multiple comparisons (Dunnett). ****P<0.0001.

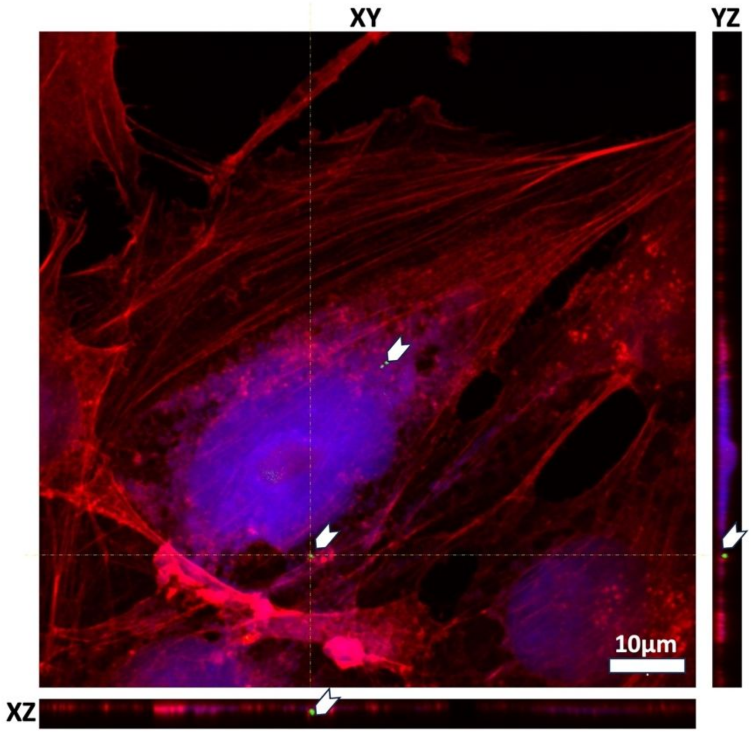

Figure S6. Confocal image and side projections of microglia cells incubated with 1μg mL $^{-1}$ of FITC–MSiNS-1. Merged images of the 3 channels (Scale bar; 10 μm). Blue: nucleus (DAPI), green: MSiNS-1 (FITC) and red: F-actin (cytopainter F).

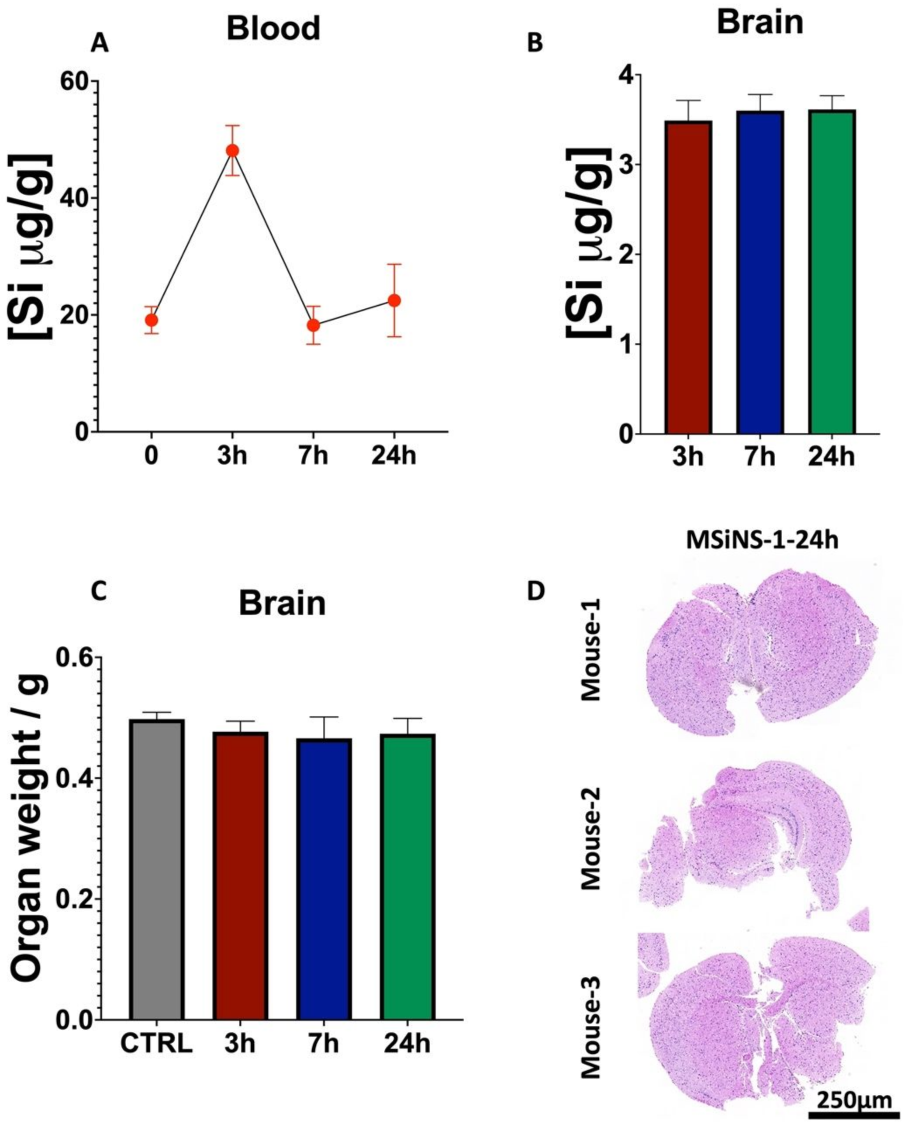

Figure S7. Quantification of silicon in blood and brain tissues and acute toxicity post IV dosing of MSiNS-1 at 10 mg/kg formulated in 0.5% methyl cellulose/water. Silicon concentration (μg/g) in A) blood and B) brain at 0, 3, 7 and 24h post dosing; (n=3). C) Changes in the Brain weight of the brains of mice at each treatment time, CTRL= mouse not treated with MSiNS-1. D) Histopathological analysis of the brain of 3 different mice after 24h of IV treatment with MSiNS-1.

Appendix

Method to model the shape of nanoparticles.

In this method, the spherical core of the nanostar is generated by mapping a Fibonacci lattice, of N points, to a unit sphere, where N is the surface area of the sphere. The points are then scaled to the radius of the NP or core of the NS. The heights of the NS tips were measured from the tangent surface of the core to the end of the tip. In generating the NS, the frustum of the cone was extended to be flush with the spherical core. The tip was generated in vertical slices with a separation distance of 1 s between the points in the lower and upper slices. Each of these slices is populated with points that are evenly distributed along a circle, ensuring that the surface area of the cone is in accordance with the precalculated value. The average separation distance between beads on the NS surface was 0.99 σ. Tips were evenly distributed across the NS surface by generating the same Fibonacci lattice, but with N equal to the number of tips, and moving the centre of the tip to the generated points.
